# Assessing Sexual Dicromatism: The Importance of Proper Parameterization in Tetrachromatic Visual Models

**DOI:** 10.1371/journal.pone.0169810

**Published:** 2017-01-11

**Authors:** Pierre-Paul Bitton, Kevyn Janisse, Stéphanie M. Doucet

**Affiliations:** Department of Biological Sciences, University of Windsor, Windsor, Ontario, Canada; University of Sussex, UNITED KINGDOM

## Abstract

Perceptual models of animal vision have greatly contributed to our understanding of animal-animal and plant-animal communication. The receptor-noise model of color contrasts has been central to this research as it quantifies the difference between two colors for any visual system of interest. However, if the properties of the visual system are unknown, assumptions regarding parameter values must be made, generally with unknown consequences. In this study, we conduct a sensitivity analysis of the receptor-noise model using avian visual system parameters to systematically investigate the influence of variation in light environment, photoreceptor sensitivities, photoreceptor densities, and light transmission properties of the ocular media and the oil droplets. We calculated the chromatic contrast of 15 plumage patches to quantify a dichromatism score for 70 species of Galliformes, a group of birds that display a wide range of sexual dimorphism. We found that the photoreceptor densities and the wavelength of maximum sensitivity of the short-wavelength-sensitive photoreceptor 1 (SWS1) can change dichromatism scores by 50% to 100%. In contrast, the light environment, transmission properties of the oil droplets, transmission properties of the ocular media, and the peak sensitivities of the cone photoreceptors had a smaller impact on the scores. By investigating the effect of varying two or more parameters simultaneously, we further demonstrate that improper parameterization could lead to differences between calculated and actual contrasts of more than 650%. Our findings demonstrate that improper parameterization of tetrachromatic visual models can have very large effects on measures of dichromatism scores, potentially leading to erroneous inferences. We urge more complete characterization of avian retinal properties and recommend that researchers either determine whether their species of interest possess an ultraviolet or near-ultraviolet sensitive SWS1 photoreceptor, or present models for both.

## Introduction

The study of animal visual systems has greatly enhanced our understanding of visual ecology and visual communication. Modelling the sensory perception of various taxa has permitted the study of animal-animal interactions such as mate choice among color morphs in butterflies [1], the evolutionary trade-off between predator-driven crypsis and sexually-selected conspicuousness in *Dendrobates* frogs [2], the influence of insect warning coloration on the predatory behavior of foraging birds [3], and the rejection of brood parasite eggs by host species [4, 5]. Furthermore, visual modelling has been useful in studies of plant-animal interactions. These include, for example, the evolution of flower colors driven by pollinator visual systems [6], the evolution of seed color as a form of crypsis against foraging birds [7], crypsis in plants to avoid predatory herbivores [8], the ability of birds to detect and select high-lipid fruits [9, 10], and the comparative ability of dichromat and trichromat primates in discriminating fruit from leaves [11]. Central to these studies is the concept of color discrimination thresholds limited by photoreceptor noise [12, 13]. This psychophysiological model of chromatic vision quantifies perception in color differences [14–16], with the caveat that the light environment and the properties of the visual system, which are central to the model, are well understood.

Visual system properties needed to produce informative visual models must include the photoreceptor sensitivities, transmission properties of the ocular media, the relative photoreceptor noise levels (approximated by relative photoreceptor densities), and for animals such as birds, lizards, and turtles, the properties of the photoreceptor-specific oil droplets which act as filters and micro-lenses [17–20]. While molecular methods and microspectrophotometry are increasingly used to determine the physical properties of visual systems (e.g., [21, 22]), complete characterizations are available for relatively few species. Furthermore, groups of closely related species have rarely been compared (see [23] for an exception). To circumvent these lack of data, research using birds as models have relied on ‘average’ visual system information (calculations and data for ultraviolet sensitive and ultraviolet insensitive eye types presented in [24]), or used parameters from closely related species. Initial comparative analyses assumed a strong association between visual systems and phylogeny [25–27], but recent studies have shown that this is not always the case. Changes between ultraviolet sensitive (UVS) and violet sensitive (VS) eye types have occurred several times in some Orders (e.g., Passeriformes and Charadriiformes: [28]), and both UVS and VS eye types can be present within the same family (e.g., Maluridae: [29]). Furthermore, the very basic organizations of visual systems can differ among and within Orders. For example, a large majority of birds characterized to date possess four distinctive single cone types (Short-wavelength-sensitive 1 –SWS1, Short-wavelength-sensitive 2 –SWS2, Medium-wavelength-sensitive–MWS, and Long-wavelength-sensitive–LWS, (e.g., [30, 31]), but exceptions have been found. The tawny owl (*Strix aluco* Linnaeus), and possibly other nocturnal raptors, lacks the UVS–VS SWS1 pigment and is therefore trichromatic [32, 33]. In contrast, the bobolink (*Dolichonyx oryzivorus* Linnaeus; Passeriformes) possesses five distinct classes of single cones: four narrowly tuned photoreceptors and one broadband photoreceptor [34]. Clearly, not all birds share the same visual sensory experience, and the incorrect parameterization of visual models could potentially lead to erroneous conclusions for a variety of ecological questions (see [27, 35, 36] for a debate example).

The possible effects of differential visual model parameterization have been explored in a diversity of taxa. Studies comparing dicromats to trichromats [37, 38] and trichromats to tetrachromats [39, 40] have quantified how these visual systems differ in their ability to tell apart patches with different spectral properties. Others have tested the effect of different light environments [22, 41–43], photoreceptors sensitivities [22, 44], photoreceptor densities [44, 45], oil droplet characteristics [44, 46–48], ocular media [22], and receptor signal-to-noise ratio [44]. While these studies have been very informative when considered together, the use of different visual system starting points and non-standardized methods of presenting results have made it difficult to compare the relative effect of each parameter within a single context. In this study, we conduct a sensitivity analysis of the receptor-noise model by systematically testing the effect of single parameters on measures of sexual dichromatism among 70 species of Galliformes, a group characterized by extreme variation in sexual dimorphism. Furthermore, we explore the effects of also varying two or more parameters on three example species.

The receptor-noise model for color discrimination is commonly used to evaluate sexual dichromatism (differences in coloration) within species, and to characterize color divergence among closely related or incipient species (e.g., [49–51]). Sexual dichromatism scores (the sum or average of differences between corresponding male and female plumage patch colors) have been used to study the evolution of sexual dichromatism [52], the influence of sexual selection on sexual dichromatism [53, 54], the relationship between sexual dichromatism and conspicuousness [55], and factors that may account for congeneric color diversity [29]. In addition, sexual dichromatism has been used as a proxy for the intensity of sexual selection in comparative studies [54, 56]. Although it has been demonstrated that human visual assessments produce different but similar approximations of dichromatism scores compared to tetrachromatic birds [13, 40, 57, 58], the effects of parameterization of bird visual models in assessments of dichromatism scores has never been systematically determined. In this study, we calculated the chromatic sexual dichromatism scores of 15 color patches for each of 70 species of Galliformes using the receptor-noise model developed by Vorobyev and Osorio [12]. For each patch, sexual dichromatism is calculated as the just-noticeable-difference (JND) in color between males and females. We evaluated the influence of light environments, photoreceptor sensitivities, oil droplet characteristics, ocular transmission, and photoreceptor densities on chromatic contrast values. The purpose of our study was to understand the relative effect of each model parameter on overall dichromatism scores, and to guide researchers when making assumptions about visual systems in studies using visual models.

## Materials and Methods

### Spectral measurements

**Fig 1:**
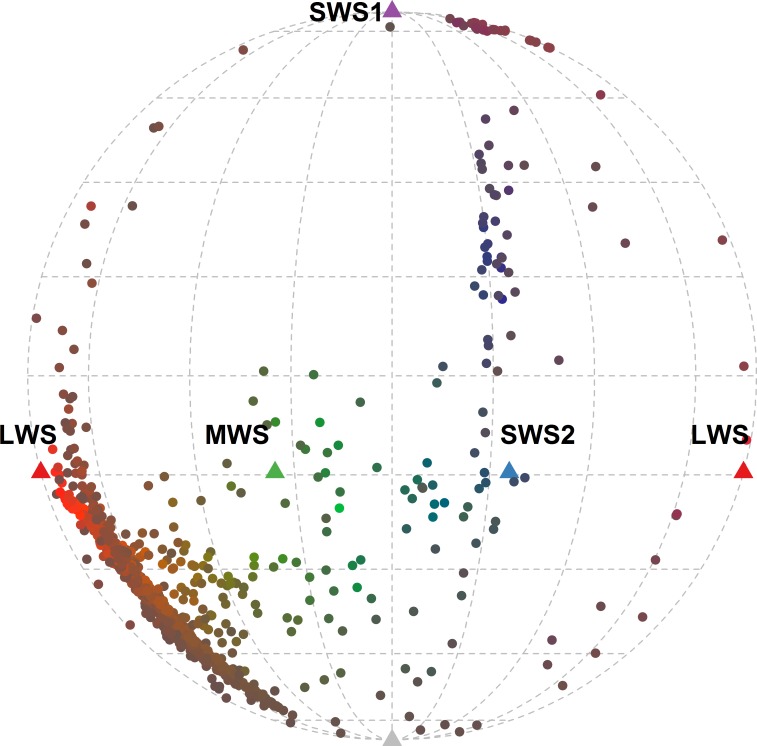
Molleweide projection of the 2100 color patches used in this study when perceived by an average UVS visual system under ideal illumination. The colors of the symbols are approximations of the colors of the patches based on a human visual system. SWS1, SWS2, MWS, and LWS refer to the ultraviolet-, short-, medium-, and long-wavelength photoreceptor, respectively.

Species in the Order Galliformes exhibit a tremendous diversity in sexual dichromatism, ranging from completely monochromatic to highly dichromatic. Across the various species in this order, feather coloration is predominantly produced by melanin pigmentation or structural colors [59], with very few plumage patches colored by carotenoid pigments [60]. We selected 70 species, most of them broadly distributed across the family Phasianidae (65 of 70 species), and measured 15 plumage patches on three males and three females of each species when available (list of species and specimen museum accession numbers in Table A in S3 File). We obtained spectral reflectance measurements using a USB 4000 spectrophotometer combined with a PX-2 Xenon light source (Ocean Optics, Dunedin, FL). We collected measurements using a bifurcated probe with a rubber stopper tip, which blocked out ambient light and maintained the probe at normal incidence and ~3 mm above the feather surface. We measured each region five times, haphazardly relocating the probe each time, and used the average of the 15 measurements (five in each of three individuals) in subsequent analyses [61]. The range of colors across the species measured covered ~58% of the gamut of structural bird colors obtained in a comprehensive survey of plumage coloration (Fig 1; [62]).

### General procedures

We tested the general influence of visual model parameterization on the mean dichromatism score of each of the 70 species, and further examined the species most affected by the different parameters. For each plumage patch, we compared the spectral reflectance of the male color to that of the female color by calculating the chromatic contrast (the difference between the colors in JNDs). We calculated the mean dichromatism score for each species as the sum of dichromatism scores across all patches divided by the number of patches (e.g., [52, 63, 64]). Some species possess non-feathered facial patches which, in museum skins, loose their color. For these species, the mean score was calculated based on the number of feathered patches. JND values smaller than 1 (i.e., non-discriminable) were given values of ‘0’ to avoid inflating the dichromatism scores based on non-detectable differences. For three of the 70 species, we then explored the multidimensional parameter space by calculating the average dichromatism scores generated by every possible combination of parameters presented in this study.

We thoroughly surveyed the literature with ISI Web of Science (accessed Dec 1^st^ 2014) for all bird studies reporting quantitatively assessed visual system parameters (e.g., using microspectrophotometry), but did not use predicted values based on gene expression (e.g., SWS1 peak sensitivity based on opsin amino acid substitution, [65]). We compiled the information available on avian photoreceptor sensitivities (Table B in S3 File), oil droplet characteristics (Table C in S3 File), transmission properties of ocular media (Table C in S3 File), and photoreceptor retinal densities (Table D in S3 File). We summarized these data separating UVS from VS eye type. We also extracted from the literature the most commonly used light environments (see below for more details).

We tested the influence of each visual model parameter by comparing dichromatism scores obtained from systematically changing the value of a single parameter. This was accomplished using the R package pavo functions (sensmodel, vismodel, and coldist; [66, 67]). Within-parameter effects were evaluated first by comparing each set of dichromatism scores against those obtained using the most commonly implemented visual models, the average VS or UVS eye type [24], followed by pairwise comparisons to find the parameter values that produced the most divergent scores. The effects of parameterization were assessed in two ways: 1) by calculating the Pearson’s correlation coefficient between the mean dichromatism scores, and 2) by comparing the ranks of the mean dichromatism scores (as per [40]). We determined how many species maintained the same rank, the mean and standard deviation of the change in rank, and the maximum change in rank. The species most affected by the changes in parameterization (largest absolute difference in mean dichromatism score and largest change in rank) were selected for further analysis. For these species, we determined the number of patches (out of 15) considered non-distinguishable under each of the two visual models, the number of patches that changed by more than 1 JND, and the maximum dichromatism score change in a single patch (in JNDs and percentage).

### Model parameterization

Average visual model–Our basis for comparisons were the two average visual models (UVS and VS eye type) presented by Endler and Mielke [24] (Table 1). In addition to the parameter values detailed in Table 1, we used the relative photoreceptor densities of the Pekin Robin (*Leiothrix lutea* Scopoli; SWS1:SWS2:MWS:LWS = 1:2:2:4) as used originally by Vorobyev and Osorio [12] and Vorobyev et al. [13], and set the Weber fraction to 0.05 for the LWS photoreceptor ([13, 68, 69]; Formulae detailed in S2 File).

**Table 1:** Parameters used to reproduce the average VS and average UVS avian visual systems presented in Endler and Mielke (2005).

Eye type	Ocular media (nm)	Parameter	UVS/VS	SWS	MWS	LWS
UVS	324					
		Peak sensitivities (nm)	367	444	501	564
		λ cut	NA†	411	511	572
		Bmid	NA	0.0278	0.023	0.022
VS	348*					
		Peak sensitivities (nm)	412	452	505	565
		λ cut	NA	447	510	570
		Bmid	NA	0.0294	0.028	0.020

These visual systems were used as the starting point for comparison of the various parameters. λ cut: Oil droplet cut-off wavelength; Bmid: Gradient of line tangent to absorbance spectrum at λ mid.

*Endler and Mielke (2005) indicate a value of 362 nm for the ocular media cut-off point but we could only reproduce the photoreceptor curves from their supplemental material when using 348 nm.

†Oil droplets associated with the SWS1 photoreceptor do not filter light between 300 and 700 nm.

Light environment–We compared the influence of the seven most commonly used environmental illuminants [70, 71]: 1) Ideal (wavelength independent), 2) Forest Shade, 3) Woodland shade, 4) Blue sky, 5) daylight D65 standard [72], 6) Woodland Gaps, and 7) Cloudy sky.

Photoreceptor sensitivities–The large majority of avian species possess four retinal cone pigments: two short-wavelength- sensitive pigments SWS1 and SWS2, one medium-wavelength-sensitive pigment MWS, and one long-wavelength-sensitive pigment LWS. The spectral sensitivity curves of these pigments can be accurately estimated from the wavelength of maximum sensitivity using a near-universal template [73]. The peak wavelength sensitivities of the four avian photoreceptors among species are correlated [17], but still allow for considerable variation in the individual sensitivity values within any given visual system. Therefore, we evaluated the influence of changes in single photoreceptor peak sensitivities, using the minimum and maximum reported for each photoreceptor type for each eye type (Table E in S3 File), and then compared visual models that expressed either all minimum or all maximum peak sensitivity values.

Oil droplets–In birds, each photoreceptor type is paired with a specific oil droplet type that acts as a cut-off filter and microlens [13, 17, 18]. SWS1 photopigments are associated with mostly transparent droplets (T type), SWS2 pigments with droplets clear in appearance (C type), MWS pigments with yellow droplets (Y type), and LWS pigments with red droplets (R types). The absorption profile of the oil droplets can be extrapolated if the wavelength at which the oil droplet transmittance equals 1/*e* (λ_o_) and the absorptivity rate of decay (b) are known. In turn, these properties can be estimated from the cut-off wavelength (λ cut) and the gradient of line tangent to the absorbance spectrum (Bmid) at the wavelength at half-maximum absorbance (λ mid), the only values that are reported in some studies of oil droplet characteristics (formulae presented in [17] and reproduced in S2 File with acronyms in S1 File). Because there is no strong relationship between a visual pigment’s peak sensitivity and the absorbance characteristics of its oil droplet (within droplet type) [17], we evaluated the influence of differences in extreme cut-off values within photopigment type first by changing single oil droplet parameters, and then by comparing visual systems with oil droplet values set with all maximums and all minimums, by eye type (Table F in S3 File). Extremes were selected based on λ cut, and actual values of either Bmid (if available from the literature) or Bmid calculated from λ mid (Table C in S3 File).

Ocular media–Similar to oil droplets, the cornea and lens act as a cut-off filters. Recent work has demonstrated that phylogeny and eye type can be used to estimate the approximate high-pass cut-off values of the ocular media in birds [22], but that variability within UVS and VS eye types, and in certain groups, is very high (e.g., waterbirds [21]). The absorption curves of ocular media in birds are all very similar and can be well approximated (function from [24] reproduced in S2 File) when the wavelength at 50% transmission (T50) is known. We evaluated the influence of varying the T50 value between 314 nm and 344 nm for UVS type eyes (the range of values known to exist for this eye type, Table C in S3 File), and between 335 nm and 395 nm in VS eye type (Table C in S3 File), using four values with equal intervals (10 nm increments among models for the UVS eye type and 20 nm for the VS eye type, Table F in S3 File).

Photoreceptor densities–The relative densities of photoreceptors vary within and among species, and even within individuals, with some evidence for bilateral asymmetry in at least two species (European starling *Sturnus vulgaris* Linnaeus and Blue tit *Cyanistes caeruleus* Linnaeus [31, 74]). Furthermore, photoreceptor densities are more heavily influenced by the ecology of the species (diet, feeding behavior, habitat) rather than phylogeny [31]. Therefore, patterns of receptor densities are difficult to predict. We tested the influence of this parameter by selecting nine different photoreceptor densities reported in a variety of species (not including the original 1:2:2:4; see Table G in S3 File for species and reasoning behind inclusion). So that the models would be comparable, we maintained the Weber fraction of the most abundant photoreceptor at 0.05 by using a different standard deviation of noise in a receptor for each model [13, 75].

Model visual systems–The physical properties of visual systems have been completely characterized in only eight species (See Results). These systems were compared to each other and to either the average UVS or VS system based on the peak wavelength sensitivity of their SWS1 photoreceptor. Because they are the most commonly used sets of parameter values, we also compared the dichromatism scores generated using the average UVS and VS eye type visual models. For all models we used a Weber fraction of 0.05 (the most commonly used), which was empirically determined for the LWS photoreceptor of *Leiothrix lutea* [68], and confirmed though behavioral tests in domesticated chicken *Gallus gallus* Linnaeus [69].

Multidimensional parameter space–To explore the effects of modifying one or more than one parameters at the same time, we selected three of the 70 species (*Tetraogallus tibetanus*, *Arborophila torqueola*, and *Lophophorus impejanus*) and calculated their average dichromatism scores for all possible combinations of parameter conditions detailed above. We selected these species using three criteria: 1) perceived by an average visual system of a VS eye-type, these species’ dichromatism score represent a low score species, an average score species, and a high score species, 2) they do not possess any bare parts, and 3) some of the patches (using the average visual system of a VS eye type) have values below 1 JND. For each eye type, the parameters comprised seven light environments including the commonly used ‘ideal’ illuminant, four T50 ocular media values, 11 combinations of spectral sensitivity values including the ‘average’ passerine, nine combinations of photoreceptor oil droplet values including the ‘average’ passerine, and nine photoreceptor density ratios including the ‘average’ passerine. We generated the mean dichromatism scores for all 24 948 possible permutations for each eye type, and present descriptive statistics for mean scores and the number of patches with scores above 1 JND.

## Results

**Fig 2:**
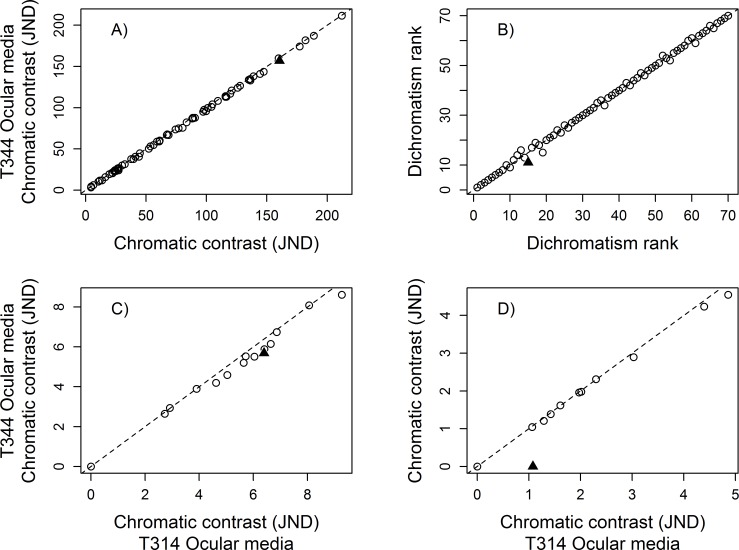
Effect of the ocular media parameter on calculated sexual dichromatism scores. Comparison of the A) mean sexual dichromatism scores (in JNDs), and B) sexual dichromatism ranks of 70 galliform species for a UVS eye type with different ocular media transmission. Values on the x-axis were generated by setting the T50 of the ocular media at 314 nm (see formulae in S2 File) and the values on the y-axis were generated by setting the T50 of the ocular media at 344 nm. The solid triangle symbol in A) identifies the species that experienced the greatest change in mean dichromatism score, and the greatest change in rank in B). The sexual dichromatism score of each patch of the species highlighted in A), under the two sets of parameters, are presented in C). The sexual dichromatism score of each patch of the species highlighted in B), under the two sets of parameters, are presented in D). In C) and D), the solid triangle symbol identifies the patch that experienced the greatest change in dichromatism score. Dashed lines represent 1:1 reference line.

**Fig 3:**
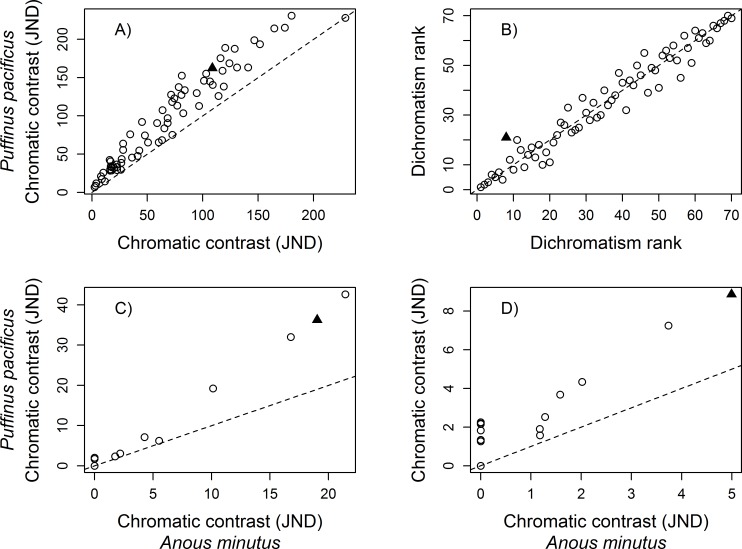
Effect of the photoreceptor density parameter on calculated sexual dichromatism scores. Comparison of A) the mean sexual dichromatism scores (in JNDs), and B) ranks of 70 galliform species for a UVS eye type with different photoreceptor densities. Values on the x-axis were generated using the parameters associated with the ‘average UVS eye-type’ visual system but with the photoreceptor densities found in *Anous minutus* (SWS1–1.00, SWS2–9.59, MWS– 16.82, LWS– 14.29); values on the y-axis were generated with the ‘average UVS eye-type’ visual system but with the photoreceptor densities found in *Puffinus pacificus* (SWS1–1.00, SWS2–0.68, MWS– 1.04, LWS– 1.44; see Methods). The solid triangle symbol in A) identifies the species that experienced the greatest change in mean dichromatism score, and the greatest change in rank in B). The sexual dichromatism score of each patch of the species highlighted in A), under the two sets of parameters, are presented in C). The sexual dichromatism score of each patch of the species highlighted in B), under the two sets of parameters, are presented in D). In C) and D), the solid triangle symbol identifies the patch that experienced the greatest change in dichromatism score. Dashed lines represent 1:1 reference line.

**Fig 4:**
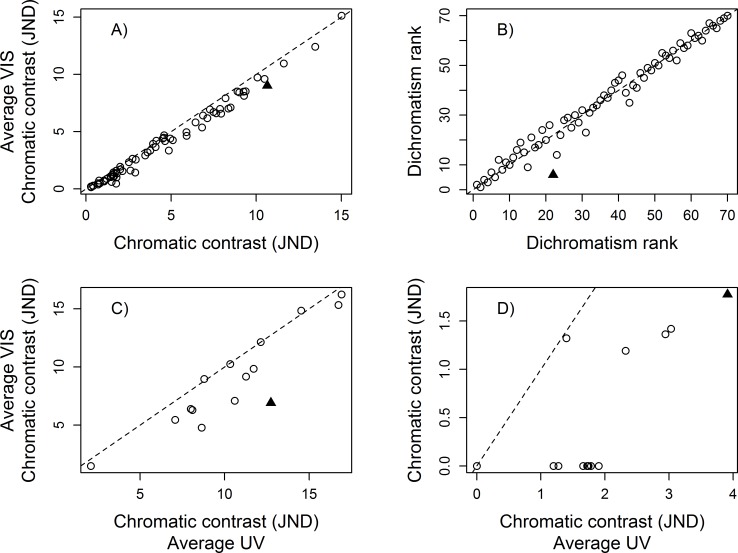
Effect of using either the UVS or VS average eye-type on calculated sexual dichromatism scores. Comparison of A) the mean sexual dichromatism scores (in JNDs), and B) ranks of 70 galliform species contrasting the two most commonly used bird visual systems. Values on the x-axis were generated using the parameters associated with the ‘average UVS eye-type’ visual system; values on the y-axis were generated with the ‘average VS eye-type’ visual system (see Methods). The solid triangle symbol in A) identifies the species that experienced the greatest change in mean dichromatism score, and the greatest change in rank in B). The sexual dichromatism score of each patch of the species highlighted in A), under the two sets of parameters, are presented in C). The sexual dichromatism score of each patch of the species highlighted in B), under the two sets of parameters, are presented in D). In C) and D), the solid triangle symbol identifies the patch that experienced the greatest change in dichromatism score. Dashed lines represent 1:1 reference line.

The mean dichromatism scores across the 70 species were always highly correlated regardless of which visual system parameter was altered (Summary in Table 2; details in Tables H–M in S3 File). Pearson’s coefficient (r) of the largest differences, within parameter, ranged from 0.9998 when contrasting the extreme T50 ocular media values for the UVS eye type (Table 2, Fig 2A), to 0.9791 when contrasting the photoreceptor densities of *Anous minutus* Boie (Black noddy) to those of *Puffinus pacificus* Gmelin (Wedged-tailed shearwater) in a UVS eye type (Table 2, Fig 3A). However, there was considerable variation in the number of species that maintained the same dichromatism rank, the mean rank change, and the maximum rank change. For example, the extreme T50 ocular media values for the UVS eye type had a moderate effect on the overall ranks and rank changes (45 out of 70 with equal rank, a mean rank change of 0.51, and maximum rank change of 4; Fig 2B), but the differences in photoreceptor densities in the UVS eye type had a large impact on the mean dichromatism ranks (only 8 out of 70 with equal rank, a mean rank change of 3.11, and maximum rank change of 12; Fig 3B). Comparisons of the dichromatism scores calculated with the commonly-used average UVS and average VS eye sets of conditions produced one of the largest difference in rank scores (Table 2, Fig 4B). Overall, photoreceptor densities and the variation among model systems had the largest influence on the mean dichromatism scores and rank differences. Variation in the transmission properties of the ocular media (within eye type) and the light environment had less influence on the mean dichromatism scores and on the ranks of species (Table 2).

**Table 2:** Summary of the visual system comparisons that generated the largest differences, based on the lowest Pearson’s r value, in the mean dichromatism scores of 70 species of the Order Galliformes.

Parameter	Eye type	Conditions	Pearson’s r	Equal rank	Rank change	Rank SD	Max change
Light environment	UVS	Ideal vs D65	0.9991	30	1.03	1.57	10
	VS	Ideal vs D65	0.9994	36	0.77	1.23	8
Photoreceptor λmax	UVS	All Max vs All Min	0.9986	27	1.03	1.20	5
	VS	All Max vs All Min	0.9963	17	1.54	1.71	9
Oil droplet cut-off value	UVS	R Min vs All Max	0.9979	27	1.00	1.13	6
	VS	R Max vs All Min	0.9947	21	1.26	1.20	5
Ocular media T50 values	UVS	T314 vs T344	0.9998	45	0.51	0.85	4
	VS	T335 vs T395	0.9991	28	1.06	1.46	9
Photoreceptor densities	UVS	*A*. *minutus* vs *P*. *pacificus*	0.9791	8	3.11	2.87	12
	VS	*A*. *minutus* vs *P*. *pacificus*	0.9846	6	2.63	2.07	10
Model systems	-	Average UVS vs Average VS	0.9929	15	2.34	2.66	16
	-	*P*. *cristatus* vs *S*. *vulgaris*	0.9881	8	3.00	3.06	16

Values reported describe how many species (out of 70) were assigned the same rank in the comparison (Equal rank), the average rank change (Rank change), the standard deviation of rank change (Rank SD), and the maximum rank change (Max change). See Materials and Methods, Results, and Supporting Information sections for more details.

Analyzing the scores of individual species most affected by changes in visual model values, based on the largest differences in mean dichromatism scores, we found that large changes in plumage patch chromatic contrast (in JND) can occur when manipulating single parameters (Table 3). The maximum changes in percentage JNDs ranged from 12.5% (0.99 JNDs) when comparing the extreme T50 ocular media values for the UVS eye type, to 84.16% (5.82 JNDs) when comparing an UVS eye type to a VS eye type (Fig 4C). Different model systems also had large effects on the chromatic contrast of some patches (Table 3). The species most affected in their mean dichromatism scores almost always had the same number of non-dichromatic patches under the two sets of conditions (comparing column 1 and column 2 of Table 3), but the number of patches that changed by more than 1 JND (column 3 Table 3) varied considerably across parameters. For the T50 ocular media value in a UVS eye, none of the patches changed by at least 1 JND. In contrast, 14 patches changed by more than 1 JND when comparing the photoreceptor densities of *A*. *minutus* and *P*. *pacificus* using the UVS eye type. Overall, individual patches were mostly influenced by condition changes in the photoreceptor densities and the variation among model systems. We also found large differences in patch dichromatism scores when comparing the average UVS and average VS eye type (Fig 4C). Variation in light environments, photoreceptor sensitivities, and ocular media values had relatively small but notable effects on the dichromatism scores of some patches (Table 3).

**Table 3:** Summary of the changes in dichromatism score of the species most affected by changes in the sensory exprience, based on the absolute largest difference in mean dichromatism score.

Parameter	Eye type	Conditions	Condition 1 (< 1 JND)	Condition 2 (< 1 JND)	> 1 JND change	Max change (JNDs)	Max change (%)
Light environment	UVS	Ideal vs D65	4	4	4	1.30	25.95
	VS	Ideal vs D65	10	14	4	1.22	NA
Photoreceptor λmax	UVS	All Max vs All Min	4	4	5	1.34	22.95
	VS	All Max vs All Min	3	3	5	2.75	17.77
Oil droplet cut-off value	UVS	R Min vs All Max	1	1	9	4.73	28.53
	VS	R Max vs All Min	1	1	10	7.16	41.64
Ocular media T50 values	UVS	T314 vs T344	0	0	0	0.99	12.48
	VS	T335 vs T395	9	14	5	1.33	NA
Photoreceptor densities	UVS	*A*. *minutus* vs *P*. *pacificus*	1	1	14	7.16	54.13
	VS	*A*. *minutus* vs *P*. *pacificus*	3	3	10	10.47	49.19
Model systems	-	Average UVS vs Average VS	0	0	9	5.82	84.16
	-	*P*. *cristatus* vs *S*. *vulgaris*	1	1	11	8.43	32.12

Values reported describe the number of patches (out of 15) without any discernable dichromatism (just-noticeable-differences < 1) under the first set of conditions (Condition 1: < 1 JND) and under the second set of conditions (Condition 2: < 1 JND), the number of patches that changed by more than 1 JND when comparing the first and second set of conditions (> 1 JND change), the maximum dichromatism value change for a single patch (Maximum change in JND) and its percentage change (Maximum change in percentage); NA values indicate that the score under one of the conditions is < 1 JNDs. See Methods and Results for more details.

When analyzing the scores of individual species most affected by changes in visual model values, based on the largest differences in dichromatism ranks, we found that large differences in ranks were associated with changes in the number of distinguishable patches (number of patches with 0 JNDs) under the two conditions (Table 4). The parameter conditions that generated the largest changes in ranks also had large differences in the number of distinguishable patches under the different sets of parameters. For example, the extreme T50 ocular media values for the UVS eye type differed by only a single patch that changed by more than 1 JND (4 patches with JND < 1 for T50 of 314 nm compared to 5 for T50 of 344 nm). In contrast, comparison of the *A*. *minutus* (8 patches with JND < 1) and *P*. *pacificus* (1 patch with JND < 1) photoreceptor densities in a UVS eye type generated 12 patches with changes in JND > 1 (Table 4). Contrary to species most affected when comparing mean dichromatism scores, we did not find large changes in plumage patch chromatic contrast (in JND) when manipulating single parameters. Indeed, none of the patches differed by more than 3.6 JNDs across all parameter values. Differences in light environment, photoreceptor sensitivities, and ocular media values generally had the smallest effects on dichromatism scores of individual patches in species with the greatest change in dichromatism ranks; changes in photoreceptor densities, and comparisons of model systems generated the largest effects (Table 4).

**Table 4:** Summary of the changes in dichromatism score of the species most affected by changes in the sensory exprience, based on the largest difference in rank.

Parameter	Eye type	Conditions	Condition 1 (< 1 JND)	Condition 2 (< 1 JND)	> 1 JND change	Max change (JNDs)	Max change (%)
Light environment	UVS	Ideal vs D65	2	4	2	1.27	NA
	VS	Ideal vs D65	10	4	4	1.22	NA
Photoreceptor λmax	UVS	All Max vs All Min	2	0	2	1.12	NA
	VS	All Max vs All Min	13	8	5	1.43	NA
Oil droplet cut-off value	UVS	R Min vs All Max	9	7	2	1.04	NA
	VS	R Max vs All Min	4	6	2	1.02	NA
Ocular media T50 values	UVS	T314 vs T344	4	5	1	1.08	NA
	VS	T335 vs T395	9	14	5	1.33	NA
Photoreceptor densities	UVS	*A*. *minutus* vs *P*. *pacificus*	8	1	12	3.62	73.72
	VS	*A*. *minutus* vs *P*. *pacificus*	14	7	7	1.36	NA
Model systems	-	Average UVS vs Average VS	2	10	12	2.14	120.30
	-	*P*. *cristatus* vs *S*. *vulgaris*	8	2	14	2.40	136.36

Values reported describe the number of patches (out of 15) without any discernable dichromatism (just-noticeable-differences < 1) under the first set of conditions (Condition 1: < 1 JND) and under the second set of conditions (Condition 2: < 1 JND), the number of patches that changed by more than 1 JND when comparing the first and second set of conditions (> 1 JND change), the maximum dichromatism value change for a single patch (Maximum change in JND) and its percentage change (Maximum change in percentage); NA values indicate that the score under one of the conditions is < 1 JNDs. See Methods and Results for more details.

**Fig 5:**
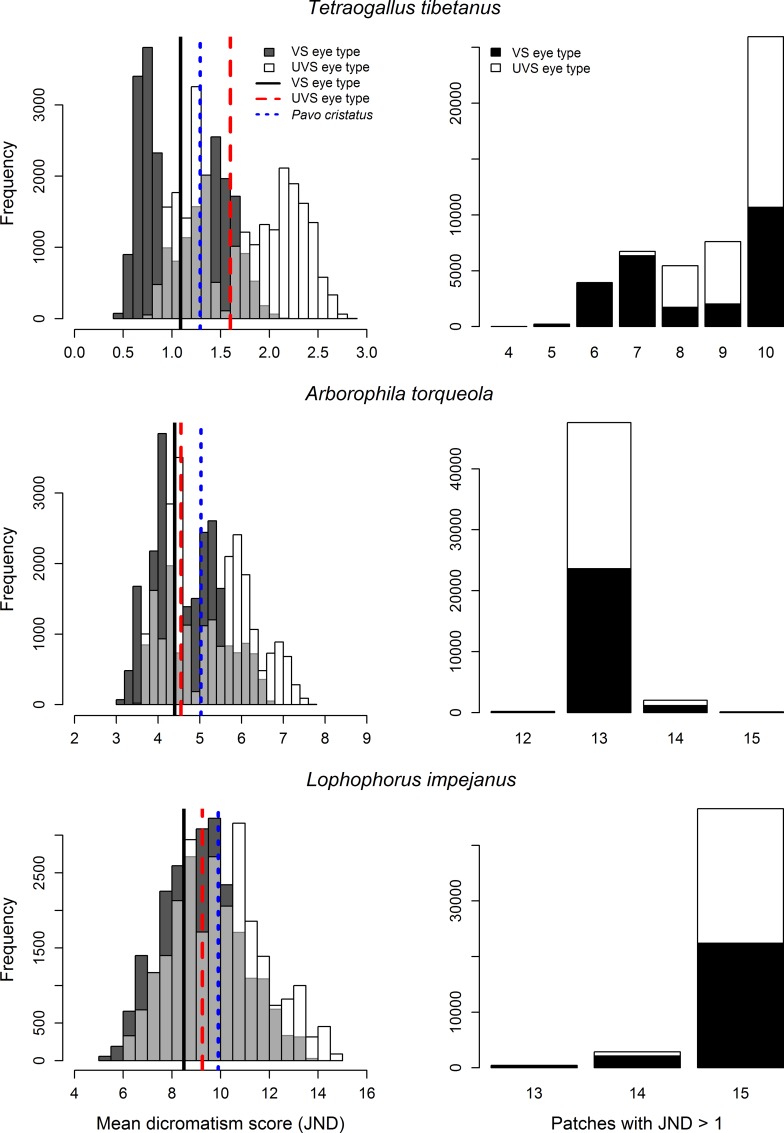
Range of sexual dichromatism scores of three galliform species obtained through exploration of the multidimensional parameter space. Distribution of mean dichromatism scores (left panels) and number of patches (out of 15) with scores greater than 1 JND (right panels), for three species, generated through permutation of all parameter conditions explored in this study (see Multidimensional parameter space section in Methods). Lines for average UVS eye type, average VS eye type, and *Pavo cristatus* indicate values calculated under an ideal illuminant.

The permutation of all conditions included in the sensitivity analyses presented above generated 24 948 dichromatism scores per species per eye type (Fig 5). For *Tetraogallus tibetanus*, a species with low dichromatism scores, the mean scores ranged from 0.41 to 2.81 JNDs when combining the UVS and VS permutation scores, a 685% difference between the lowest and highest values (Table N in S3 File). The distribution of scores for the two eye types overlapped over only 58% of their combined range, with the mean score of the UVS eye type being on average 1.51 times greater the mean VS eye type scores. For *Lophophorus impejanus*, a species with high dichromatism scores, the mean scores ranged from 5.26 to 14.77 JNDs when combining the UVS and VS permutation scores, a 281% difference between the lowest and highest values. The distribution of scores for the two eye types overlapped over 80% of their combined range, substantially more than *T*. *tibetanus*, with the mean score of the UVS eye type being on average 1.08 times greater the mean VS eye type scores. For *Arborophila torqueola*, a species with mid-range dichromatism scores, the mean scores ranged from 3.00 to 7.68 JNDs when combining the UVS and VS permutation scores, a 256% difference between the lowest and highest values. The distribution of scores for the two eye types overlapped over 70% of their combined range, with the mean score of the UVS eye type being on average 1.11 times greater than the mean VS eye type scores. In accordance with results obtained when modifying single parameters, the species with the lowest mean dichromatism score, *T*. *tibetanus* in this case, varied most in the number of patches with scores above 1 JND across all permutations (Fig 5). The number of patches above discrimination threshold ranged from four to 10 in VS eye type, and from seven to 10 in UVS eye type. The number of patches above discrimination threshold varied little for *A*. *torqueola* and *L*. *impejanus*, with a large majority of permutations indicating 13 and 15 patches, respectively, with values above 1 JND.

## Discussion

Compared to fish and invertebrates, avian visual systems exhibit considerably less variation in physical and physiological properties [71]. Nevertheless, a number of studies suggest that variation in avian visual systems appears to be adaptive. Carotenoid-based signals are aligned with cone sensitivities across species of the Passerida clade of passerine birds [76]; the expression of opsin genes are associated with sexual dichromatism in New World warblers [77]; the ocular media of UVS eye types allow more UV light to reach the retina than the ocular media of VS eye types [78]; and the photoreceptor densities among species seem to be ecologically relevant [30]. Other studies have failed to find alignment between the visual system and the behavior or ecology of species. Ultraviolet vision, for example, does not seem to have co-evolved with plumage coloration across most bird families [23, 79], but see [29]. Proper parameterization of visual models could be of paramount importance when investigating spectral tuning and the evolution of visual systems in birds. This also applies to studies of sexual dichomatism, which have been used to investigate, among other topics, the influence of sexual selection on speciation [54, 56].

In this study, we systematically compared the effects of changing single parameters in visual models on mean dichromatism scores, and ranks of dichromatism scores, in 70 species of Galliformes. We found high correlations between the mean dichromatism scores regardless of the differences among the models, but found that the rank of individual species could be greatly affected. These results imply that even though parameterization has little effect on the general distribution of the mean dichromatism scores, changes in visual system assumptions can have large effects on the relative position of the mean dichromatism scores of species in relation to one another. Such variation could be important when comparing dichromatism scores to other ecological or life history traits. Furthermore, certain parameters had a large influence on the number of patches considered above discrimination threshold (JND > 1), and the dichromatism scores of individual patches. Consequences of incorrect parameterization could easily lead to the improper inference that species are sexually dichromatic or not. Our findings suggest that the parameterization of avian visual systems should not be trivialized. Indeed, the differences in chromatic contrast we documented between patches were in some cases as large as those reported when comparing color discrimination between trichromats and tetrachromats [40].

Light environment–Some of the earliest research aimed at assessing the importance of visual model parameters demonstrated that differences in light environments made Trinidadian guppies (*Poecilia reticulate* Peters) more conspicuous in the presence of conspecifics than in the presence of heterospecific predators [80]. The importance of this parameter was further demonstrated in behavioral trials which showed that the absence of UV wavelengths reduced the foraging efficiency of three-spined sticklebacks (*Gasterosteus aculeatus* Linnaeus; [43]). In contrast, the light environment has almost no effect on the discriminability of vole urine against the vegetation background (~4% JNDs) suggesting little influence on the behavior of foraging raptors [22]. The results from these studies, and others (e.g., [41, 42, 81]), indicate that the importance of the light environment in visual models is context dependent. In our study, differences in light environment had relatively small but non-negligible effects on the mean dichromatism scores and ranks in Galliformes. The JNDs of some of the patches changed by more than 25%, differences considerably larger than those modelled by Lind et al. [22]. Our largest observed differences included the daylight 65 (D65) illuminant for both UVS and VS eye types (Table 4). This commonly-modelled light environment is rich in blue (but not UV) wavelength and is likely to influence colors rich in red and UV wavelength such as those produced by some carotenoids [82]. Also of great importance is the consideration that color constancy mechanisms, approximated by the von Kries correction in the receptor noise model, limit the influence of the light environment on the perceived contrast between color patches. Behavioural tests [83] and modelling experiments [84] in vertebrates have shown that chromatic adaptation can work over large changes in ambient light environments.

Photoreceptor sensitivities–In a test of parameterization effects on models in foraging raptors, Lind et al. [22] reported chromatic differences of ~16% ± 12% JNDs when comparing a range of photoreceptor sensitivities. These results were obtained by changing the SWS1 and SWS2 photoreceptor by 10 nm towards short-wavelength sensitivity and the LWS by 10 nm towards long-wavelength sensitivity. Our general results support these findings and also demonstrate that differences greater than ~23% are possible. These values were obtained by comparing all minimum-shifted and all maximum-shifted photoreceptor sensitivities (Table C in S3 File). However, changes in the sensitivity of single photoreceptors within eye type, even to the extreme known values across birds, had very little influence on dichromatism scores (Table I in S3 File). These results also support work presented by Lind and Kelber [44], which found little influence of photoreceptor sensitivity in modelling chromatic differences between four colors (peak wavelength at 350, 450, 500, and 650 nm) against a green background. Overall, the parameterization of photoreceptor sensitivities should only have consequential influences on chromatic contrast calculations when all sensitivities are wrongfully shifted in the same direction (all towards short- or long-wavelengths) or when SWS and LWS photoreceptors are shifted in opposite directions. In contrast, changes in single photoreceptor sensitivities generally have limited effects on calculated JND scores, but some consequential effects on dichromatism ranks.

Oil droplet cut-off value–Variation in the transmission cut-off values of oil droplets had similar but potentially slightly larger influence on dichromatism scores than variation in photoreceptor sensitivities. Our results demonstrate that differences greater than 41% (> 7 JNDs in this case) in single patch chromatic contrasts are possible. Lind and Kelber [44] also demonstrated the importance of this parameter. In a behavioural experiment comparing the measured and predicted visual sensitivities in domestic pigeons (*Columba livia*), the match between visual models and behavioral results in tests of hue discriminability improved tremendously by shifting the absorbance curves of oil droplets by 10 nm in their models. Oil droplet properties of the avian eye should perhaps be given greater attention. Indeed, considerable variation has been found within species, both among individuals and between the sexes [85]. Modelling of within-species differences suggest chromatic contrast differences as large as ~30% JNDs in some parts of the visual spectrum [48], sufficient to influence the perspective of the receivers, and potentially affecting foraging and mate choice behaviors. Furthermore, recent experiments have revealed that dietary carotenoid content can influence the transmission properties of oil droplets in double cones, indicating condition-based within-species variation in visual properties and the potential of diet to influence color vision [86]. To date, the influence of variation in oil droplet characteristics on color discrimination have only been modelled, never behaviorally tested.

Ocular media–The only other study to have explicitly modelled the influence of ocular media on chromatic contrasts found this parameter rather inconsequential [44]. Our overall results (Table 2) agree with these findings but highlighted that this parameter is perhaps most important when modelling VS eye types, not UVS eye types (Table 4). Differences in this parameter will only influence the perception of UV-rich colors which are common in fruits and feathers that contain carotenoids [82, 87], vole-urine used by foraging raptors to assess prey density [22, 88], and some structural colors (e.g.,[89–91]). Particular consideration to this parameter should be made when modelling color discrimination of these UV rich colors.

Photoreceptor densities–The photoreceptor density was the single most important parameter in our models both in terms of changes in mean dichromatism ranks and chromatic contrast of individual patches (Tables 2 and 4). This occurs because the Weber fraction is proportional to the inverse of the square root of the photoreceptor ratio (see formula in supplemental material in [12, 75]), and has a linear relationship with JNDs. For example, a dichromatic score of 6 JNDs for a Weber fraction of 0.05 will be reduced to 3 JNDs with a Weber faction of 0.10, and further reduced to 1.5 JNDs with a Weber fraction of 0.20. It is perhaps not surprising then, that photoreceptor density has such a large influence on chromatic contrast thresholds. Individual patches changed by as much as 10.5 JNDs (~50%), values in the range of to those presented by Lind and Kelber [44], which demonstrates the importance of this visual system trait. Differences in photoreceptor densities are likely to have large consequences on among-species ability to discriminate between similar colors and, as for variation in oil droplet absorbance profiles, within-species variation may be of consequence as well. For example, differences in densities among house sparrows (*Passer domesticus* Linnaeus) generated chromatic contrast differences of ~16% (> 3 JNDs) when evaluating the perception of the white wing bars against the brown wing background coloration [45]. Even if these plumage patches likely differ more in the achromatic component of the signal, these chromatic differences may still have implications for mate choice and agonistic interactions. Because the characterization of complete visual systems requires specialized equipment and skills, and the sacrifice of animals, our knowledge of photoreceptor densities come from relatively few studies (see Hart [30] for the majority of species characterized to date). Future research on the physical properties of avian retinas should obtain as much information as possible, including counts of different photoreceptor types. Just as importantly, the assumptions that 1) relative receptor noise is truly proportional to receptor abundance and that 2) receptor noise is independent of receptor type need to be rigorously tested. These data have the potential to make large contributions to our understanding of the visual ecology of birds.

Model systems–Within eye types, there was relatively small difference in mean dichromatism scores among the model visual systems. However, our results suggest that one of the most influential parameters of visual models is whether a species possesses a UVS or VS eye type (Tables 2–4, Table M in S3 File). This is of particular importance since these are the two most commonly used sets of parameters in avian visual modelling. Because there was a strong belief in phylogenetic inertia in eye type (e.g., [25, 26]), studies have usually modelled a single eye type (exceptions include: [92–94]). However, as demonstrated by Renoult et al.[27], using the wrong eye type can entirely alter the conclusions of a study. Fortunately, determination of a species’ eye type does not require microspectrophotometry, as opsin gene sequences can be used to estimate sensitivity [33]. Even though not all SWS1 opsin gene sequence variations have been compared to measured photopigment sensitivities, the peak absorbance of short-wavelength photoreceptor can usually be estimated [28, 33]. This method is relatively rapid, inexpensive, and could easily be implemented in any molecular laboratory [33]. Alternatively, when this is not possible, both eye types could be modelled when the eye type is not known.

Without a doubt, sensory perceptions are produced by the combined physical properties of sensory systems. By calculating the range of mean dichromatism scores for all parameter combinations, we demonstrated that misinformed parameterization could lead to very different conclusions. In certain instances, two or more parameters may cancel each other out (e.g., a short-wavelength shifted SWS1 value matched with a long-wavelength shifted ocular media value), but other combinations of incorrect parameters could dramatically alter calculated values. For example, a short-wavelength-shifted SWS1 photoreceptor sensitivity value matched with a photoreceptor density ratio that favours discriminability in the short wavelengths could mistakenly modify a UV-insensitive visual system to one that can detect small color differences in the UV range. In extreme cases, as demonstrated by the permutation scores calculated for *T*. *tibetanus*, improper parameterization could lead to differences between calculated and actual contrasts of more than 650%. As for errors in single parameters, errors in multiparameter space are more likely to affect the scores of species with low dichromatism scores, and models that generate values near threshold should be interpreted with caution. Minimizing uncertainty in the most influential parameters, namely photoreceptor densities and peak sensitivity of the SWS1 photoreceptor, should increase confidence in the calculated dichromatism scores.

Caution is also needed when making inferences regarding large contrast values in units of just-noticeable-differences. Indeed, the primary function of the receptor noise model is to computationally determine discrimination thresholds, based on a psychophysical model [12]. As such, the meaning of supra-threshold distances is generally unknown. Is a color patch differing by 5 JNDs as discriminable from a reference as a patch differing by 7 JNDs? Following Fechner’s assumption of additivity, large colorspace should be possible to model (discussed in [95, 96] among others). Furthermore, the only study investigating a behavioral response (in this case the visual attention reflex) in relation to perceptually large color distances found that just-noticeable-differences, as calculated by the receptor noise model, are effective in describing colors separated by large distances in visual space [97]. Nevertheless, because the evidence is sparse, very large differences in units of JND should be interpreted with prudence. It is also important to consider that animals do not perceive individual patches in isolation [24] and that the contrasts among several color patches may be evaluated differently than the sum of its parts, concepts that are currently being investigated [98]. Whether or not the mean (or sum) of dichromatism scores are meaningful beyond a discrimination threshold will need further study.

Overall, our results suggest that a single parameter can have large influences on avian dichromatism scores calculated by combining the chromatic contrast of several plumage patches, and the rank position of a species and/or their color patches in relation to one another. Furthermore, we demonstrated that contrasts calculated by varying multiple parameters can generate extreme discrepancies between estimated dichromatism scores. To improve the reliability of avian visual models, information about photoreceptor densities and the sensitivity of the SWS1 photoreceptor should be investigated when possible. Because sequencing the SWS1 gene is cost-effective, we recommend that researchers modelling avian visual systems determine, at least, whether their species of interest possess a UVS or VS eye type. Alternatively, results should present analyses from both eye types to confirm that the findings are robust. When little visual system information is available for a species of interest, in birds and other systems, reporting the results of sensitivity analyses would help strengthen the inferences presented.

## Supporting Information

S1 File List of Abbreviations. (DOCX)

S2 File Formulae. (DOCX)

S3 File Supporting information. (DOCX)

S4 File Average spectral curves from 3 males, and 3 females, from 70 species of Galliform, 15 plumage patches per species. (CSV)

S5 File Species by plumage patch matrix indicating which patches are bare (patches not used in analyses). (CSV)

## References

[1] Limeri LB, Morehouse NI. Sensory limitations and the maintenance of colour polymorphisms: viewing the 'alba' female polymorphism through the visual system of male *Colias* butterflies. Funct Ecol. 2014;28(5):1197–207.

[2] Willink B, Bolanos F, Prohl H. Conspicuous displays in cryptic males of a polytypic poison-dart frog. Behav Ecol Sociobiol. 2014;68(2):249–61.

[3] Cibulková A, Veselý P, Fuchs R. Importance of conspicuous colours in warning signals: the great tit's (*Parus major*) point of view. Evol Ecol. 2014;28(3):427–39.

[4] Croston R, Hauber ME. Spectral tuning and perceptual differences do not explain the rejection of brood parasitic eggs by American robins (*Turdus migratorius*). Behav Ecol Sociobiol. 2014;68(3):351–62.

[5] Spottiswoode CN, Stevens M. Visual modeling shows that avian host parents use multiple visual cues in rejecting parasitic eggs. P Natl Acad Sci USA. 2010;107(19):8672–6. 10.1073/pnas.0910486107 PMC2889299 20421497

[6] Muchhala N, Johnsen S, Smith SD. Competition for hummingbird pollination shapes flower color variation in Andean Solanaceae. Evolution. 2014;68(8):2275–86. 10.1111/evo.12441 24766107

[7] Lev-Yadun S, Ne'eman G. Bimodal colour pattern of individual *Pinus halepensis* Mill. seeds: a new type of crypsis. Biol J Linn Soc. 2013;109(2):271–8.

[8] Niu Y, Chen G, Peng DL, Song B, Yang Y, Li ZM, et al Grey leaves in an alpine plant: a cryptic colouration to avoid attack? New Phytol. 2014;203(3):953–63. 10.1111/nph.12834 24800901

[9] Schaefer HM, Valido A, Jordano P. Birds see the true colours of fruits to live off the fat of the land. P Roy Soc B-Biol Sci. 2014;281(1777). 10.1098/rspb.2013.2516 PMC3896014 24403330

[10] Cazetta E, Schaefer HM, Galetti M. Why are fruits colorful? The relative importance of achromatic and chromatic contrasts for detection by birds. Evol Ecol. 2009;23(2):233–44.

[11] Melin AD, Hiramatsu C, Parr NA, Matsushita Y, Kawamura S, Fedigan LM. The behavioral ecology of color vision: Considering fruit conspicuity, detection distance and dietary importance. Int J Primatol. 2014;35(1):258–87.

[12] Vorobyev M, Osorio D. Receptor noise as a determinant of colour thresholds. P Roy Soc B-Biol Sci. 1998;265(1394):351–8. 10.1098/rspb.1998.0302 PMC1688899 9523436

[13] Vorobyev M, Osorio D, Bennett ATD, Marshall NJ, Cuthill IC. Tetrachromacy, oil droplets and bird plumage colours. J Comp Physiol A. 1998;183(5):621–33. 9839454 10.1007/s003590050286

[14] Osorio D, Vorobyev M. Photoreceptor spectral sensitivities in terrestrial animals: adaptations for luminance and colour vision. P Roy Soc B-Biol Sci. 2005;272(1574):1745–52. 10.1098/rspb.2005.3156 PMC1559864 16096084

[15] Kelber A, Vorobyev M, Osorio D. Animal colour vision—behavioural tests and physiological concepts. Biol Rev. 2003;78(1):81–118. 12620062 10.1017/s1464793102005985

[16] Osorio D, Vorobyev M. A review of the evolution of animal colour vision and visual communication signals. Vision Res. 2008;48(20):2042–51. 10.1016/j.visres.2008.06.018 18627773

[17] Hart NS, Vorobyev M. Modelling oil droplet absorption spectra and spectral sensitivities of bird cone photoreceptors. J Comp Physiol A. 2005;191(4):381–92. 10.1007/s00359-004-0595-3 15711964

[18] Stavenga DG, Wilts BD. Oil droplets of bird eyes: microlenses acting as spectral filters. Philos T R Soc B. 2014;369(1636). 10.1098/rstb.2013.0041 PMC3886329 24395968

[19] Wilby D, Toomey MB, Olsson P, Frederiksen R, Cornwall MC, Oulton R, et al Optics of cone photoreceptors in the chicken (*Gallus gallus domesticus*). Journal of the Royal Society Interface. 2015;12(111). 10.1098/rsif.2015.0591 PMC4614498 26423439

[20] Loew ER, Fleishman LJ, Foster RG, Provencio I. Visual pigments and oil droplets in diurnal lizards: a comparative study of Caribbean anoles. J Exp Biol. 2002;205(7):927–38. 11916989 10.1242/jeb.205.7.927

[21] Håstad O, Partridge JC, Ödeen A. Ultraviolet photopigment sensitivity and ocular media transmittance in gulls, with an evolutionary perspective. J Comp Physiol A. 2009;195(6):585–90. 10.1007/s00359-009-0433-8 19308422

[22] Lind O, Mitkus M, Olsson P, Kelber A. Ultraviolet sensitivity and colour vision in raptor foraging. J Exp Biol. 2013;216(10):1819–26. 23785106 10.1242/jeb.082834

[23] Coyle BJ, Hart NS, Carleton KL, Borgia G. Limited variation in visual sensitivity among bowerbird species suggests that there is no link between spectral tuning and variation in display colouration. J Exp Biol. 2012;215(7):1090–105. 22399654 10.1242/jeb.062224

[24] Endler JA, Mielke PW. Comparing entire colour patterns as birds see them. Biol J Linn Soc. 2005;86(4):405–31.

[25] Eaton MD. Human vision fails to distinguish widespread sexual dichromatism among sexually "monochromatic" birds. P Natl Acad Sci USA. 2005;102(31):10942–6. 10.1073/pnas.0501891102 PMC1182419 16033870

[26] Bridge ES, Hylton J, Eaton MD, Gamble L, Schoech SJ. Cryptic plumage signaling in *Aphelocoma* Scrub-Jays. J Ornithol. 2008;149(1):123–30.

[27] Renoult JP, Courtiol A, Kjellberg F. When assumptions on visual system evolution matter: nestling colouration and parental visual performance in birds. J Evolution Biol. 2010;23(1):220–5. 10.1111/j.1420-9101.2009.01885.x 19895654

[28] Ödeen A, Håstad O. The phylogenetic distribution of ultraviolet sensitivity in birds. BMC Evol Biol. 2013;13. 23394614 10.1186/1471-2148-13-36 PMC3637589

[29] Ödeen A, Pruett-Jones S, Driskell AC, Armenta JK, Håstad O. Multiple shifts between violet and ultraviolet vision in a family of passerine birds with associated changes in plumage coloration. P Roy Soc B-Biol Sci. 2012;279(1732):1269–76. 10.1098/rspb.2011.1777 PMC3282372 21976683

[30] Hart NS. The visual ecology of avian photoreceptors. Prog Retin Eye Res. 2001;20(5):675–703. 11470455 10.1016/s1350-9462(01)00009-x

[31] Hart NS. Variations in cone photoreceptor abundance and the visual ecology of birds. J Comp Physiol A. 2001;187(9):685–97. 11778831 10.1007/s00359-001-0240-3

[32] Bowmaker JK, Martin GR. Visual pigments and color-vision in a nocturnal bird, *Strix aluco* (Tawny Owl). Vision Res. 1978;18(9):1125–30. 716232 10.1016/0042-6989(78)90095-0

[33] Ödeen A, Håstad O. Complex distribution of avian color vision systems revealed by sequencing the SWS1 opsin from total DNA. Mol Biol Evol. 2003;20(6):855–61. 10.1093/molbev/msg108 12716987

[34] Beason RC, Loew ER. Visual pigment and oil droplet characteristics of the bobolink (*Dolichonyx oryzivorus*), a new world migratory bird. Vision Res. 2008;48(1):1–8. 10.1016/j.visres.2007.10.006 18054982

[35] Avilés JM, Soler JJ. Nestling coloration is adjusted to parent visual performance in altricial birds irrespective of assumptions on vision system for Laniidae and owls, a reply to Renoult et al. J Evolution Biol. 2010;23(1):226–30. 10.1111/j.1420-9101.2009.01890.x 19895653

[36] Avilés JM, Soler JJ. Nestling colouration is adjusted to parent visual performance in altricial birds. J Evolution Biol. 2009;22(2):376–86. 10.1111/j.1420-9101.2008.01655.x 19196385

[37] Perini ES, Pessoa VF, Pessoa DMA. Detection of fruit by the Cerrado's marmoset (*Callithrix penicillata*): Modeling color signals for different background scenarios and ambient light intensities. J Exp Zool Part A. 2009;311a(4):289–302. 10.1002/jez.531 19296489

[38] Cheney KL, Marshall NJ. Mimicry in coral reef fish: how accurate is this deception in terms of color and luminance? Behav Ecol. 2009;20(3):459–68.

[39] Siddiqi A, Cronin TW, Loew ER, Vorobyev M, Summers K. Interspecific and intraspecific views of color signals in the strawberry poison frog *Dendrobates pumilio*. J Exp Biol. 2004;207(14):2471–85. 15184519 10.1242/jeb.01047

[40] Håstad O, Ödeen A. Different ranking of avian colors predicted by modeling of retinal function in humans and birds. Am Nat. 2008;171(6):831–8. 10.1086/587529 18429674

[41] Avilés JM. Egg colour mimicry in the common cuckoo *Cuculus canorus* as revealed by modelling host retinal function. P Roy Soc B-Biol Sci. 2008;275(1649):2345–52. 10.1098/rspb.2008.0720 PMC2603233 18595836

[42] Holveck MJ, Doutrelant C, Guerreiro R, Perret P, Gomez D, Gregoire A. Can eggs in a cavity be a female secondary sexual signal? Male nest visits and modelling of egg visual discrimination in blue tits. Biol Letters. 2010;6(4):453–7. 10.1098/rsbl.2009.1044 PMC2936209 20164078

[43] Rick IP, Bloemker D, Bakker TCM. Spectral composition and visual foraging in the three-spined stickleback (Gasterosteidae: *Gasterosteus aculeatus* L.): elucidating the role of ultraviolet wavelengths. Biol J Linn Soc. 2012;105(2):359–68.

[44] Lind O, Kelber A. Avian colour vision: Effects of variation in receptor sensitivity and noise data on model predictions as compared to behavioural results. Vision Res. 2009;49(15):1939–47. 10.1016/j.visres.2009.05.003 19460402

[45] Ensminger AL, Fernández-Juricic E. Individual variation in cone photoreceptor density in House sparrows: Implications for between-individual differences in visual resolution and chromatic contrast. Plos One. 2014;9(11). 10.1371/journal.pone.0111854 PMC4221115 25372039

[46] Goldsmith TH, Butler BK. The roles of receptor noise and cone oil droplets in the photopic spectral sensitivity of the budgerigar, *Melopsittacus undulatus*. J Comp Physiol A. 2003;189(2):135–42. 10.1007/s00359-002-0385-8 12607042

[47] Vorobyev M. Coloured oil droplets enhance colour discrimination. P Roy Soc B-Biol Sci. 2003;270(1521):1255–61. 10.1098/rspb.2003.2381 PMC1691374 12816638

[48] Ronald KL, Fernández-Juricic E, Lucas JR. Taking the sensory approach: how individual differences in sensory perception can influence mate choice. Anim Behav. 2012;84(6):1283–94.

[49] Burns KJ, Shultz AJ. Widespread cryptic dichromatism and ultraviolet reflectance in the largest radiation of Neotropical songbirds: Implications of accounting for avian vision in the study of plumage evolution. Auk. 2012;129(2):211–21.

[50] Delhey K, Peters A. Quantifying variability of avian colours: Are signalling traits more variable? Plos One. 2008;3(2). 10.1371/journal.pone.0001689 PMC2253496 18301766

[51] Macías-Sánchez E, Martinez JG, Avilés JM, Soler M. Sexual differences in colour and size in the Great Spotted Cuckoo *Clamator glandarius*. Ibis. 2013;155(3):605–10.

[52] Price JJ, Eaton MD. Reconstructing the evolution of sexual dichromatism: Current color diversity does not reflect past rates of male and female change. Evolution. 2014;68(7):2026–37. 10.1111/evo.12417 24689951

[53] Pérez i de Lanuza G, Font E, Monterde JL. Using visual modelling to study the evolution of lizard coloration: sexual selection drives the evolution of sexual dichromatism in lacertids. J Evolution Biol. 2013;26(8):1826–35. 10.1111/jeb.12185 23848517

[54] Huang HT, Rabosky DL. Sexual selection and diversification: Reexamining the correlation between dichromatism and speciation rate in birds. Am Nat. 2014;184(5):E101–E14. 10.1086/678054 25325752

[55] Doucet SM, Mennill DJ, Hill GE. The evolution of signal design in manakin plumage ornaments. Am Nat. 2007;169(1):S62–S80. 29517930 10.1086/510162

[56] Seddon N, Botero CA, Tobias JA, Dunn PO, MacGregor HEA, Rubenstein DR, et al Sexual selection accelerates signal evolution during speciation in birds. P Roy Soc B-Biol Sci. 2013;280(1766). 10.1098/rspb.2013.1065 PMC3730587 23864596

[57] Armenta JK, Dunn PO, Whittingham LA. Quantifying avian sexual dichromatism: a comparison of methods. J Exp Biol. 2008;211(15):2423–30. 18626076 10.1242/jeb.013094

[58] Seddon N, Tobias JA, Eaton M, Ödeen A. Human vision can provide a valid proxy for avian perception of sexual dichromatism. Auk. 2010;127(2):283–92.

[59] Durrer H, Villiger W. Schillerstruktur des Kongopfaus (*Afropavo congensis* Chapin, 1936) im Elektronenmikroskop. Journal für Ornithologie 1975;116(1):94–102.

[60] Thomas DB, McGraw KJ, Butler MW, Carrano MT, Madden O, James HF. Ancient origins and multiple appearances of carotenoid-pigmented feathers in birds. P Roy Soc B-Biol Sci. 2014;281(1788). 10.1098/rspb.2014.0806 PMC4083795 24966316

[61] Dalrymple RL, Hui FKC, Flores-Moreno H, Kemp DJ, Moles AT. Roses are red, violets are blue—so how much replication should you do? An assessment of variation in the colour of flowers and birds. Biol J Linn Soc. 2015;114(1):69–81.

[62] Stoddard MC, Prum RO. How colorful are birds? Evolution of the avian plumage color gamut. Behav Ecol. 2011;22(5):1042–52.

[63] Badyaev AV, Hill GE. Evolution of sexual dichromatism: contribution of carotenoid- versus melanin-based coloration. Biol J Linn Soc. 2000;69(2):153–72.

[64] Dunn PO, Armenta JK, Whittingham LA. Natural and sexual selection act on different types of variation in avian plumage color. Science Advances. 2015;1:e1400155 10.1126/sciadv.1400155 26601146 PMC4643820

[65] Ödeen A, Hart NS, Håstad O. Assessing the use of genomic DNA as a predictor of the maximum absorbance wavelength of avian SWS1 opsin visual pigments. J Comp Physiol A. 2009;195(2):167–73. 10.1007/s00359-008-0395-2 19048261

[66] Maia R, Eliason CM, Bitton PP, Doucet SM, Shawkey MD. pavo: an R package for the analysis, visualization and organization of spectral data. Methods in Ecology and Evolution. 2013;4(10):906–13.

[67] R Development Core Team. R: A language and environment for statistical computing. Vienna, Austria: 2016.

[68] Maier EJ. Spectral sensitivities including the ultraviolet of the passeriform bird *Leiothrix lutea*. Journal of Comparative Physiology A-Sensory Neural and Behavioral Physiology. 1992;170(6):709–14.

[69] Olsson P, Lind O, Kelber A. Bird colour vision: behavioural thresholds reveal receptor noise. J Exp Biol. 2015;218(2):184–93. 25609782 10.1242/jeb.111187

[70] Endler JA. The color of light in forests and its implications. Ecol Monogr. 1993;63(1):1–27.

[71] Cronin TW, Johnsen S, Marshall NJ, Warrant EJ. Visual ecology US: Princeton University Press; 2014.

[72] Jn Schanda, International Commission on Illumination. Colorimetry: understanding the CIE system Vienna, Austria: CIE/Commission internationale de l'eclairage; Wiley-Interscience; 2007. xxix, 459 p. p.

[73] Govardovskii VI, Fyhrquist N, Reuter T, Kuzmin DG, Donner K. In search of the visual pigment template. Visual Neurosci. 2000;17(4):509–28. 10.1017/s0952523800174036 11016572

[74] Hart NS, Partridge JC, Cuthill IC. Visual pigments, oil droplets and cone photoreceptor distribution in the European starling (*Sturnus vulgaris*). J Exp Biol. 1998;201(9):1433–46. 9547323 10.1242/jeb.201.9.1433

[75] Vorobyev M, Brandt R, Peitsch D, Laughlin SB, Menzel R. Colour thresholds and receptor noise: behaviour and physiology compared. Vision Res. 2001;41(5):639–53. 11226508 10.1016/s0042-6989(00)00288-1

[76] Bleiweiss R. Physical alignments between plumage carotenoid spectra and cone sensitivities in ultraviolet-sensitive (UVS) birds (Passerida: Passeriformes). Evol Biol. 2014;41(3):404–24.

[77] Bloch NI. Evolution of opsin expression in birds driven by sexual selection and habitat. P Roy Soc B-Biol Sci. 2015;282(1798). 10.1098/rspb.2014.2321 PMC4262183 25429020

[78] Lind O, Mitkus M, Olsson P, Kelber A. Ultraviolet vision in birds: the importance of transparent eye media. P Roy Soc B-Biol Sci. 2014;281(1774). 10.1098/rspb.2013.2209 PMC3843830 24258716

[79] Lind O, Delhey K. Visual modelling suggests a weak relationship between the evolution of ultraviolet vision and plumage coloration in birds. J Evolution Biol. 2015;28(3):715–22. 10.1111/jeb.12595 25664902

[80] Endler JA. Variation in the appearance of guppy color patterns to guppies and their predators under different visual conditions. Vision Res. 1991;31(3):587–608. 1843763 10.1016/0042-6989(91)90109-i

[81] Avilés JM, Soler JJ, Hart NS. Sexual selection based on egg colour: physiological models and egg discrimination experiments in a cavity-nesting bird. Behav Ecol Sociobiol. 2011;65(9):1721–30.

[82] Britton G, Liaaen-Jensen S, Pfander H. Carotenoids. Basel; Boston: Birkhäuser Verlag; 1995.

[83] Dörr S, Neumeyer C. Color constancy in goldfish: the limits. Journal of Comparative Physiology a-Sensory Neural and Behavioral Physiology. 2000;186(9):885–96. 10.1007/s003590000141 11085641

[84] Wilkins L, Marshall NJ, Johnsen S, Osorio D. Modelling fish colour constancy, and the implications for vision and signalling in water. J Exp Biol. 2016:jeb-139147. 10.1242/jeb.139147 27045090

[85] Knott B, Bowmaker JK, Berg ML, Bennett ATD. Absorbance of retinal oil droplets of the budgerigar: sex, spatial and plumage morph-related variation. J Comp Physiol A. 2012;198(1):43–51. 10.1007/s00359-011-0684-z 21979102

[86] Knott B, Berg ML, Morgan ER, Buchanan KL, Bowmaker JK, Bennett ATD. Avian retinal oil droplets: dietary manipulation of colour vision? P Roy Soc B-Biol Sci. 2010;277(1683):953–62. 10.1098/rspb.2009.1805 PMC2842729 19939843

[87] McGraw KJ. Mechanics of carotenoid-based coloration In: Hill GE, McGraw KJ, editors. Bird coloration: Mechanisms and Measurements. 1. Cambridge, Massachusetts: Harvard University Press; 2006 p. 243–94.

[88] Viitala J, Korpimäki E, Palokangas P, Koivula M. Attraction of kestrels to vole scent marks visible in ultraviolet-light. Nature. 1995;373(6513):425–7.

[89] Andersson S, Ornborg J, Andersson M. Ultraviolet sexual dimorphism and assortative mating in blue tits. P Roy Soc B-Biol Sci. 1998;265(1395):445–50.

[90] Hunt S, Bennett ATD, Cuthill IC, Griffiths R. Blue tits are ultraviolet tits. P Roy Soc B-Biol Sci. 1998;265(1395):451–5.

[91] Doucet SM, Montgomerie R. Multiple sexual ornaments in satin bowerbirds: ultraviolet plumage and bowers signal different aspects of male quality. Behav Ecol. 2003;14(4):503–9.

[92] Uy JAC, Endler JA. Modification of the visual background increases the conspicuousness of golden-collared manakin displays. Behav Ecol. 2004;15(6):1003–10.

[93] Gomez D, Théry M. Simultaneous crypsis and conspicuousness in color patterns: Comparative analysis of a neotropical rainforest bird community. Am Nat. 2007;169(1):S42–S61. 29517929 10.1086/510138

[94] Langmore NE, Stevens M, Maurer G, Kilner RM. Are dark cuckoo eggs cryptic in host nests? Anim Behav. 2009;78(2):461–8.

[95] Renoult JP, Kelber A, Schaefer HM. Colour spaces in ecology and evolutionary biology. Biol Rev. 2015. 10.1111/brv.12230 26468059

[96] Gn Wyszecki, Stiles WS. Color science: concepts and methods, quantitative data, and formulae Wiley classics library ed. New York: John Wiley & Sons; 2000. xv, 950 p. p.

[97] Fleishman LJ, Perez CW, Yeo AI, Cummings KJ, Dick S, Almonte E. Perceptual distance between colored stimuli in the lizard *Anolis sagrei*: comparing visual system models to empirical results. Behav Ecol Sociobiol. 2016;70(4):541–55.

[98] Cole GL, Endler JA. Variable environmental effects on a multicomponent sexually selected trait. Am Nat. 2015;185(4):452–68. 10.1086/680022 25811082

