# Supplementary material for: Assessing Sexual Dicromatism: The Importance of Proper Parameterization in Tetrachromatic Visual Models
Source: PLoS One. 2017 Jan 11;12(1):e0169810. doi: 10.1371/journal.pone.0169810 (PMC5226829; doi:10.1371/journal.pone.0169810)
Supplement: S1 File — (DOCX) [file pone.0169810.s001.docx]

**S1 Supplemental Material – List of Abbreviations**

| **Context** |  |  |
| --- | --- | --- |
|  |  |  |
| General |  |  |
|  | JND | Just-noticeable difference |
|  | UVS | Ultraviolet-sensitive photoreceptor class |
|  | VS | Violet-sensitive photoreceptor class |
|  | λ | Wavelength |
|  | ΔS | Cromatic contrast between two stimuli |
|  | SWS | Short-wavelength-sensitive photoreceptor class |
|  | MWS | Medium-wavelength-sensitive photoreceptor class |
|  | LWS | Long-wavelength-sensitive photoreceptor class |
|  | λmax | Wavelength at maximum absorbance |
|  |  |  |
| Oil droplets and ocular media |  |  |
|  | λcut | Oil droplet cut-off wavelength |
|  | λmid | Wavelength at half-maximum absorbance (oil droplet) |
|  | λ_o_ | Wavelength at which oil droplet transmittance is 1/*e* |
|  | b | Transmission rate of decay (oil droplet) |
|  | Bmid | Gradient of line tangent to absorbance spectrum at λmid (oil droplet) |
|  | T50 | Wavelength at 50% transmission of the ocular media |
|  | Te | Absorbance curve of the ocular media |
|  |  |  |
| Visual models |  |  |
|  | Q | Quantum catch of a photoreceptor class (relative) |
|  | S(λ) | Sensitivity spectrum of a photoreceptor class (normalized) |
|  | I(λ) | Irradiance spectrum |
|  | R(λ) | Reflectance spectrum |
|  | Δf | Contrast in a photoreceptor class |
|  | σ | Standard deviation of noise in a photoreceptor |
|  | ω | Weber fraction |
|  |  |  |
