# Supplementary material for: Assessing Sexual Dicromatism: The Importance of Proper Parameterization in Tetrachromatic Visual Models
Source: PLoS One. 2017 Jan 11;12(1):e0169810. doi: 10.1371/journal.pone.0169810 (PMC5226829; doi:10.1371/journal.pone.0169810)
Supplement: S2 File — (DOCX) [file pone.0169810.s002.docx]

**S2 Supplemental Material - Formulae**

*Chromatic contrast for tetrachromatic visual system*

The receptor-noise model (Vorobyev and Osorio 1998) states that the color stimulus of surface x is defined by the quantum catch of each photoreceptor class I such that:

$$Q_{i,x}= \int_{{}_{\min}}^{{}_{\max}} R_{x}\left( \right)S_{i}()I\left( \right)d()$$

where λ is wavelength, $R_{x}\left( \right)$ is the wavelength-specific reflectance spectrum of the object $x$, $S_{i}()$ is the wavelength-specific spectral sensitivity of receptor $i$, and $I\left( \right)$ is the wavelength-specific spectrum of ambient light. Integration in this study was calculated over the visible spectrum of birds, from 300 to 700 nm.

The relationship between the quantum catch of two stimuli (a and b) for photoreceptor class i is:

$$f_{i}=\ln\left( Q_{i,a} \right)-\ln\left( Q_{i,b} \right)=ln\left( \frac{Q_{i,a}}{Q_{i,b}} \right)$$

The standard deviation of the noise of a single photoreceptor cell is represented by $\sigma_{i}$. The effect of this noise on color perception decreases with increase in the proportion of photoreceptors of a given class, such that the Weber fraction (ω) a given class of photoreceptors is:

$$\omega_{i}= \sigma_{i}/\sqrt{{}_{i}}$$

where ${}_{i}$ is the density of photoreceptors of type $i$ relative to the UVS densities, and $e_{i}$ is the noise of type $i$ of the four photoreceptor classes. All relative photoreceptor densities are calculated in relation to the UVS cone density.

Incorporating the noise and the difference in quantum catch between two stimuli, the square of chromatic contrast ($S$) of two stimuli in a tetrachromatic system is given by:

${(S)}^{2}=\left( {(\omega_{1}\omega_{2})}^{2}{(f_{4}-f_{3})}^{2}+{(\omega_{1}\omega_{3})}^{2}{(f_{4}-f_{2})}^{2}+{(\omega_{1}\omega_{4})}^{2}{(f_{3}-f_{2})}^{2}+{(\omega_{2}\omega_{3})}^{2}{(f_{4}-f_{1})}^{2}+{(\omega_{2}\omega_{4})}^{2}{(f_{3}-f_{1})}^{2}+{(\omega_{3}\omega_{4})}^{2}{(f_{2}-f_{1})}^{2} \right)/\left( \left( \omega_{1}\omega_{2}\omega_{3} \right)^{2}+\left( \omega_{1}\omega_{2}\omega_{4} \right)^{2}+\left( \omega_{1}\omega_{3}\omega_{4} \right)^{2}+\left( \omega_{2}\omega_{3}\omega_{4} \right)^{2} \right)$

Chromatic contrast is in units of just noticeable difference (or JND), representing chromatic discriminability.

*Ocular media*

The absorbance curve ($T_{e}\left( \lambda\right))$of the ocular media can be approximated using:

$$T_{e}\left( \lambda\right)=ln(8.928\times{10}^{-13}\lambda^{5} - 2.595\times{10}^{-9}\lambda^{4} +3.006\times{10}^{-6}\lambda^{3} - 0.001736\lambda^{2} + 0.5013\lambda-55.56)$$

Formula optimized for λT50 = 335.2; curves for other T50 are λ-axis shifted following Endler and Mielke (2005).

*Oil filter*

The average visual systems defined by Endler and Mielke (2005) use λ˳ (wavelength at which oil droplet transmittance is 1/e) and *b* (rate of decay) to calculate absorbance curves for C, Y, and R types. The R package pavo (Maia et al. 2013) uses λcut (cut-off wavelength) and the gradient of line tangent to the absorbance spectrum at the wavelength at half-maximum absorbance (Bmid) because λcut is the most commonly reported value in microspectrophotometry studies (e.g., Hart and Vorobyev 2005).

When λcut and Bmid were not provided, they were calculated using the following equations (from Hart and Vorobyev 2005):

$$\lambda cut= \lambda_{o}-\left( \frac{0.37}{Bmid} \right)$$

$$Bmid= \frac{b}{2.89}$$

For species for which *b* was not available we estimated Bmid:

$$Bmid=0.5\div(\lambda mid- \lambda cut)$$
