# Supplementary material for: Assessing Sexual Dicromatism: The Importance of Proper Parameterization in Tetrachromatic Visual Models
Source: PLoS One. 2017 Jan 11;12(1):e0169810. doi: 10.1371/journal.pone.0169810 (PMC5226829; doi:10.1371/journal.pone.0169810)
Supplement: S3 File — (DOCX) [file pone.0169810.s003.docx]

**S3 Supplemental Material – Supporting information**

**Table A** Catalogue numbers for all specimens measured. AMNH = American Museum of Natural History, New York; FMNH = Field Museum of Natural History, Chicago; ROM: Royal Ontario Museum, Toronto; UMMZ = University of Michigan Museum of Zoology, Ann Arbor.

| Scientific name | Common name | Male 1 | Male 2 | Male 3 | Female 1 | Female 2 | Female 3 |
| --- | --- | --- | --- | --- | --- | --- | --- |
| Acryllium vulturinum | Vulturine Guineafowl | FMNH_192683 | ROM_34.9.6.1 | FMNH_405746 | ROM_33.6.20.1 | FMNH_192686 | FMNH_406226 |
| Afropavo congoensis | Congo Peafowl | AMNH_763938 | AMNH_305691 | AMNH_763937 | AMNH_763941 | AMNH_305693 | AMNH_305692 |
| Agriocharis ocellata | Ocellated Turkey | UMMZ_95038 | FMNH_40755 | FMNH_13216 | FMNH_120862 | ROM_37192 | AMNH_61172 |
| Alectoris barbara barbara | Barbary Partridge | FMNH_404276 | FMNH_407988 | FMNH_406930 | ROM_33.9.1.125 | FMNH_404277 | FMNH_411638 |
| Alectoris graeca | Rock Partridge | ROM_91811 | ROM_36988 | ROM_146344 | ROM_146345 | ROM_146343 | FMNH_415114 |
| Alectoris rufa | Red-legged Partridge | FMNH_408741 | FMNH_408731 | FMNH_408743 | FMNH_408740 | FMNH_409848 | FMNH_408742 |
| Alectura lathami | Australian Brush-turkey | FMNH_415316 | FMNH_415320 | FMNH_417123 | FMNH_400782 | AMNH_539307 | AMNH_539306 |
| Arborophila rufogularis | Rufous-throated Hill Partridge | ROM_37049 | ROM_37050 | ROM_37048 | FMNH_84360 | FMNH_84357 | FMNH_217900 |
| Arborophila torqueola torqueola | Common Hill-partridge | ROM_37045 | ROM_37046 | ROM_37044 | ROM_37047 | FMNH_84368 | FMNH_426050 |
| Argusianus argus | Great Argus | FMNH_414261 | ROM_26510 | ROM_37157 | FMNH_414262 | FMNH_40724 | AMNH_544057 |
| Bambusicola fytchii | Mountain Bamboo-partridge | UMMZ_140962 | UMMZ_140961 | UMMZ_140960 | UMMZ_140940 | UMMZ_140915 | UMMZ_140932 |
| Bambusicola thoracica | Chinese Bamboo-partridge | UMMZ_57477 | ROM_29067 | FMNH_404463 | UMMZ_57478 | FMNH_404472 | FMNH_406543 |
| Bonasa (Tetrastes) bonasia | Hazel Grouse | ROM_36721 | FMNH_414729 | FMNH_412918 | ROM_36722 | FMNH_416633 | FMNH_412919 |
| Bonasa umbellus | Ruffed Grouse | FMNH_131131 | ROM_36770 | ROM_29214 | ROM_80226 | ROM_145996 | ROM_36772 |
| Catreus wallichi | Cheer Pheasant | FMNH_426070 | ROM_37101 | ROM_01.10.1.18 | FMNH_96808 | ROM_67550 | FMNH_16224 |
| Chrysolophus amherstiae | Lady Amherst’ Pheasant | FMNH_410562 | ROM_23.2.27.1 | ROM_68495 | FMNH_111885 | FMNH_67905 | FMNH_408997 |
| Chrysolophus pictus | Golden Pheasant | FMNH_109179 | FMNH_88551 | UMMZ_84354 | UMMZ_54313 | ROM_69042 | ROM_28221 |
| Coturnix coturnix | Common Quail | ROM_75193 | ROM_75194 | ROM_33.9.1.130 | ROM_81613 | ROM_91.11.1.900 | ROM_37019 |
| Coturnix japonica | Japanese Quail | ROM_37022 | FMNH_406545 | FMNH_411130 | ROM_37023 | FMNH_419107 | FMNH_405133 |
| Crax rubra | Great Curassow | FMNH_418523 | ROM_112814 | FMNH_15452 | ROM_36563 | FMNH_411741 | FMNH_411742 |
| Crossoptilon auritum | Blue Eared-pheasant | ROM_35.11.15.6 | ROM_66902 | FMNH_109187 | FMNH_109188 | FMNH_410201 | - |
| Crossoptilon mantchuricum | Brown Eared-pheasant | ROM_21.3.6.2 | ROM_34.3.23.5 | ROM_37080 | FMNH_392224 | ROM_22.12.15.1 | AMNH_543113 |
| Francolinus francolinus | Black Francolin | FMNH_413367 | ROM_36995 | FMNH_420353 | FMNH_420345 | FMNH_420336 | FMNH_420351 |
| Francolinus pondicerianus | Grey Francolin | FMNH_410845 | FMNH_410848 | FMNH_414052 | FMNH_414055 | FMNH_414054 | FMNH_414053 |
| Francolinus squmatus | Scaly Francolin | FMNH_423915 | FMNH_417588 | FMNH_417590 | FMNH_403913 | FMNH_423907 | FMNH_417587 |
| Francolinus swainsonii | Swainson’s Francolin | ROM_121044 | FMNH_410600 | FMNH_423156 | ROM_121043 | ROM_91212 | FMNH_486021 |
| Gallus gallus | Red Junglefowl | FMNH_420755 | FMNH_400745 | FMNH_420759 | FMNH_420737 | FMNH_420742 | FMNH_420770 |
| Gallus lafayettei | Ceylon Junglefowl | FMNH_401144 | FMNH_422530 | FMNH_401145 | FMNH_422528 | AMNH_543371 | AMNH_203777 |
| Gallus sonneratii | Grey Junglefowl | FMNH_414948 | FMNH_414949 | FMNH_420789 | FMNH_414947 | FMNH_420785 | FMNH_420802 |
| Gallus varius | Green Junglefowl | FMNH_405246 | FMNH_405166 | FMNH_406655 | FMNH_406656 | FMNH_405247 | AMNH_543402 |
| Ithaginis cruentus | Blood Pheasant | FMNH_109175 | FMNH_109176 | FMNH_403880 | FMNH_404607 | FMNH_403882 | FMNH_109177 |
| Lophophorus impejanus | Himalayan Monal | FMNH_84350 | ROM_01.10.1.37 | ROM_37078 | FMNH_84351 | UMMZ_234309 | ROM_37079 |
| Lophophorus lhuysii | Chinese Monal | FMNH_109196 | FMNH_88542 | FMNH_88543 | FMNH_88544 | AMNH_423702 | AMNH_543110 |
| Lophophorus sclateri | Sclater's Monal | AMNH_543103 | AMNH_543104 | AMNH_543108 | FMNH_97920 | AMNH_543107 | - |
| Lophura edwardsi | Edward’s Pheasant | FMNH_76414 | AMNH_348673 | - | UMMZ_119960 | ROM_3510222 | - |
| Lophura leucomelana | Kalij Pheasant | FMNH_210931 | ROM_26812 | ROM_29.1.20.1 | FMNH_210937 | FMNH_217897 | FMNH_210934 |
| Lophura nycthemera | Silver Pheasant | FMNH_408966 | ROM_36.2.25.4 | ROM_24112 | ROM_1822810 | ROM_19481 | FMNH_408967 |
| Lophura swinhoii | Swinhoe’s Pheasant | FMNH_405134 | ROM_32029 | ROM_35.12.17.6 | UMMZ_119958 | ROM_3471898 | FMNH_405135 |
| Meleagris gallopavo | Wild Turkey | FMNH_93516 | ROM_37176 | ROM_31973 | FMNH_160406 | ROM_34.5.8.3 | ROM_37181 |
| Numida meleagris | Helmeted Guineafowl | ROM_33.9.1.141 | ROM_114469 | FMNH_405724 | FMNH_405726 | FMNH_405719 | FMNH_192654 |
| Ortalis vetula | Plain Chachalaca | ROM_36570 | ROM_36571 | ROM_81801 | ROM_36572 | ROM_81800 | FMNH_409461 |
| Pavo cristatus | Indian Peafowl | ROM_26.3.23.144 | FMNH_421426 | ROM_37164 | FMNH_420844 | ROM_27.4.6.1 | ROM_37165 |
| Pavo muticus | Green Peafowl | FMNH_404960 | FMNH_92678 | ROM_37166 | FMNH_404962 | FMNH_405167 | FMNH_404961 |
| Perdix dauuricae | Daurian Partridge | FMNH_96819 | FMNH_406733 | FMNH_56309 | FMNH_56305 | FMNH_56312 | FMNH_56308 |
| Perdix hodgsoniae | Tibetan Partridge | FMNH_408819 | FMNH_67884 | AMNH_541962 | FMNH_408818 | FMNH_109182 | FMNH_109183 |
| Perdix perdix | Grey Partridge | ROM_80227 | ROM_74165 | ROM_146348 | ROM_36.1.4.1 | ROM_37006 | ROM_134533 |
| Phasianus colchius | Ring-necked Pheasant | FMNH_404491 | ROM_67044 | ROM_29588 | ROM_37135 | ROM_31.5.27.7 | ROM_29589 |
| Phasianus versicolor | Green Pheasant | FMNH_405140 | FMNH_405142 | FMNH_405141 | FMNH_405143 | FMNH_405138 | FMNH_405139 |
| Polyplectron bicalcaratum | Grey Peacock-pheasant | FMNH_400753 | FMNH_401330 | FMNH_415143 | FMNH_415142 | FMNH_400754 | AMNH_409108 |
| Polyplectron chalcurum | Bronze-tailed Peacock pheasant | AMNH_543975 | AMNH_543979 | AMNH_257149 | AMNH_543980 | AMNH_543981 | AMNH_543974 |
| Polyplectron emphanum | Palawan Peacock-pheasant | FMNH_426082 | FMNH_416817 | FMNH_416818 | FMNH_404223 | AMNH_544041 | AMNH_544040 |
| Polyplectron germaini | Germain's Peacock-pheasant | FMNH_89970 | AMNH_417029 | AMNH_544015 | ROM_37154 | AMNH_544017 | AMNH_544019 |
| Polyplectron inopinatum | Mountain Peacock pheasant | AMNH_543966 | AMNH_543971 | AMNH_804683 | AMNH_543969 | AMNH_203867 | - |
| Polyplectron malacense | Malaysian Peacock-pheasant | AMNH_544026 | AMNH_544022 | - | ROM_3250 | AMNH_203879 | - |
| Pucrasia macrolopha | Koklass Pheasant | FMNH_40731 | ROM_01.10.1.16 | ROM_37099 | ROM_37100 | FMNH_416216 | FMNH_416217 |
| Rheinardia ocellata | Crested Argus | FMNH_UCMROO | AMNH_258935 | AMNH_544046 | FMNH_76415 | AMNH_348697 | - |
| Syrmaticus ellioti | Elliot’s Pheasant | FMNH_96805 | ROM_68392 | ROM_95005 | ROM_34.3.7.1 | FMNH_39345 | AMNH_543904 |
| Syrmaticus humiae | Hume’s Pheasant | FMNH_415486 | FMNH_415488 | FMNH_415485 | FMNH_415492 | FMNH_415489 | FMNH_415496 |
| Syrmaticus reevesii | Reeve’s Pheasant | FMNH_88552 | UMMZ_119966 | ROM_37139 | UMMZ_119967 | ROM_34.3.7.2 | ROM_34.7.9.4 |
| Syrmaticus soemmerringii | Copper pheasant | FMNH_405149 | ROM_24.3.13.392 | ROM_37140 | ROM_98.4.8.2 | FMNH_96965 | FMNH_405147 |
| Tetrao tetrix | Eurasian Black Grouse | FMNH_67078 | ROM_2925 | ROM_3198 | ROM_9085 | ROM_146360 | FMNH_406927 |
| Tetrao urogallus | Western Capercaillie | FMNH_404587 | FMNH_67034 | ROM_36582 | FMNH_401633 | FMNH_406928 | FMNH_408409 |
| Tetraogallus himalayensis | Himalayan Snowcock | FMNH_410838 | FMNH_60618 | AMNH_804741 | ROM_36984 | FMNH_420101 | FMNH_410836 |
| Tetraogallus tibetanus | Tibetan Snowcock | FMNH_426040 | FMNH_410841 | FMNH_410842 | FMNH_420097 | FMNH_420095 | AMNH_540543 |
| Tetraophasis obscurus | Verreaux’s Monal Partridge | FMNH_410198 | FMNH_410199 | FMNH_109172 | FMNH_410196 | AMNH_540578 | AMNH_204527 |
| Tetraophasis szechenyii | Szechenyi’s Monal Partridge | FMNH_409970 | FMNH_411061 | AMNH_540588 | FMNH_67913 | AMNH_291992 | AMNH_540581 |
| Tragopan blythii | Blyth's Tragopan | FMNH_415463 | FMNH_415461 | FMNH_399352 | FMNH_415132 | FMNH_415462 | FMNH_415465 |
| Tragopan caboti | Cabot's Tragopan | FMNH_51135 | FMNH_407536 | ROM_10.2.21.24 | FMNH_51136 | FMNH_407663 | ROM_156309 |
| Tragopan satyra | Satyr Tragopan | FMNH_414243 | FMNH_84323 | FMNH_84321 | FMNH_84327 | FMNH_84325 | AMNH_817845 |
| Tragopan temminckii | Temminck’s Tragopan | FMNH_88534 | FMNH_88533 | FMNH_88537 | FMNH_88536 | FMNH_88540 | FMNH_88541 |

UCMROO = uncatalogued

**Table B** Spectral parameters of cone visual pigments in avian species separated by eye type (UVS range SWS1 or VS range SWS1).

| Order | Species | Photoreceptor *λ*max (nm) | | | | | References |
| --- | --- | --- | --- | --- | --- | --- | --- |
|  |  | UVS | VS | SWS2 | MWS | LWS |  |
|  |  |  |  |  |  |  |  |
| Anseriformes | *Anas platyrhynchos* |  | 415 | 452 | 506 | 567 | 1 |
|  | *Branta canadensis* |  | 409 | 458 | 509 | 580 | 2 |
| Apodiformes | *Sephanoides sphanoides* | 371 |  | 444 | 508 | 560 | 3 |
| Columbiformes | *Columbia livia* |  | 404 | 452 | 506 | 566 | 4 |
| Galliformes | *Coturnix coturnix* |  | 418 | 450 | 505 | 567 | 5 |
|  | *Gallus gallus* |  | 418 | 453 | 507 | 571 | 4 |
|  | *Meleagris gallopavo* |  | 420 | 460 | 505 | 563 | 6 |
|  | *Pavo cristatus* |  | 424 | 458 | 505 | 567 | 7 |
| Gruiformes | *Grus americana* |  | 404 | 450 | 499 | 561 | 8 |
| Passeriformes | *Amadia fasciata* | 370 |  | 447 | 500 | 563 | 9 |
|  | *Corvus frugilegus* |  |  |  | 497 | 565 | 10 |
|  | *Dolichonyx oryzivorus* | 372 |  |  | 505 | 564 | 11 |
|  | *Erythrura gouldiae* | 370 |  | 440 | 500 | 562 | 9 |
|  | *Leothrix lutea* | 355 |  | 454 | 499 | 568 | 12 |
|  | *Lonchura maja* | 373 |  | 446 | 500 | 562 | 9 |
|  | *Neochmia modesta* | 373 |  | 442 | 500 | 565 | 9 |
|  | *Parus caeruleus* | 372 |  | 449 | 502 | 563 | 13 |
|  | *Passer domesticus* |  |  | 445 | 503 | 563 | 14 |
|  | *Ptilonorhynchus violaceus* |  | 410 | 454 | 511 | 562 | 15 |
|  | *Serinus canaria* | 363 |  | 440 | 501 | 567 | 16 |
|  | *Spinus tristis* |  | 399 | 442 | 512 | 580 | 17 |
|  | *Sturnus vulgaris* | 362 |  | 449 | 504 | 563 | 18 |
|  | *Taeniopygia guttata* | 359 |  | 427 | 505 | 566 | 4, 19 |
|  | *Turdus merula* | 373 |  | 454 | 504 | 557 | 13 |
| Procellariformes | *Puffinus pacificus* |  | 406 | 450 | 503 | 566 | 20 |
|  | *Puffinus puffinus* |  | 402 | 452 |  |  | 4 |
| Psittaciformes | *Melopsittacus undulatus* | 371 |  | 440 | 499 | 566 | 4 |
|  | *Platycercus elegans* | 365 |  | 440 | 509 | 567 | 21 |
| Spheniciformes | *Spheniscus humboldti* |  | 403 | 450 |  | 543 | 22 |
| Strigiformes | *Strix aluco* |  |  | 463 | 503 | 555 | 23 |
| Struthioniformes | *Rhea americana* |  |  | 447 | 506 | 571 | 24 |
|  | *Struthio camelus* |  | 405 | 445 | 506 | 570 | 24 |

**Table C** Spectral absorption parameters for oil droplets and ocular media in avian eyes. See Methods for meaning of λcut, λmid and T50 values.

| Order | Species | λcut (nm) | | | λmid (nm) | | | SWS1  Pigment | OM T50 | Oil droplet reference | OM Reference |
| --- | --- | --- | --- | --- | --- | --- | --- | --- | --- | --- | --- |
|  |  | C type | Y type | R type | C type | Y type | R type |  |  |  |  |
| Accipitriformes | *Accipiter nisus* |  |  |  |  |  |  | VS | 369 |  | 25 |
|  | *Buteo buteo* |  |  |  |  |  |  | VS | 375 |  | 25 |
|  | *Milvus milvu* |  |  |  |  |  |  | VS | 394 |  | 25 |
| Anseriformes | *Anas platyrhynchos* | 445 | 506 | 561 | 459 | 521 | 585 | VS | 371 | 1 | 1 |
|  | *Branta canadensis* |  | 506 | 559 |  | 526 | 598 | VS |  | 2 |  |
| Apodiformes | *Apus apus* |  |  |  |  |  |  | VS | 388 |  | 25 |
|  | *Sephanoides sephanoides* |  |  |  |  |  |  | UVS | 315 |  | 3 |
| Charadriiformes | *Larus marinus* |  |  |  |  |  |  | UVS | 344 |  | 26 |
| Columbiformes | *Columba livia* | 448 | 514 | 586 | 470 | 542 | 613 | VS | 337 | 4 | 27 |
| Falconiformes | *Falco tinnunculus* |  |  |  |  |  |  | VS | 379 |  | 25 |
| Galliformes | *Conturnix conturnix* | 446 | 511 | 566 | 461 | 528 | 589 | VS |  | 5 |  |
|  | *Gallus gallus* | 443 | 505 | 561 | 460 | 523 | 586 | VS | 351 | 4 | 27 |
|  | *Meleagris gallopavo* |  |  |  |  |  |  | VS | 355 |  | 6 |
|  | *Pavo cristatus* | 449 | 511 | 569 | 462 | 525 | 592 | VS | 364 | 7 | 7 |
| Gruiformes | *Grus americana* | 448 | 522 | 576 |  |  |  | VS |  | 8 |  |
| Passeriformes | *Ailuroedus crassirostris* | 421 | 508 | 558 | 438 | 526 | 580 | VS | 340 | 15 | 15 |
|  | *Amadina fasciata* | 423 | 516 | 575 | 439 | 535 | 598 | UVS | 316 | 9 | 9 |
|  | *Chlamydera maculata* | 428 | 515 | 571 | 449 | 533 | 595 | VS | 351 | 15 | 15 |
|  | *Chlamydera nuchalis* | 421 | 515 | 568 | 440 | 530 | 590 | VS | 349 | 15 | 15 |
|  | *Corvus frugilegus* |  |  |  |  |  |  | VS | 365 |  | 25 |
|  | *Cyanistes (Parus) caeruleus* | 413 | 508 | 573 | 426 | 528 | 596 | UVS | 316 | 13 | 13 |
|  | *Dolichonyx oryzivorus* | 412 | 502 | 561 | 429 | 519 | 584 | UVS |  | 11 |  |
|  | *Erythrura gouldiae* | 422 | 513 | 572 | 434 | 531 | 595 | UVS | 315 | 9 | 9 |
|  | *Leothrix lutea* | 392 | 506 | 566 | 419 | 530 | 591 | UVS |  | 12 |  |
|  | *Lonchura maja* | 422 | 510 | 567 | 434 | 524 | 589 | UVS | 317 | 9 | 9 |
|  | *Neochmia modesta* | 415 | 514 | 568 | 428 | 534 | 591 | UVS | 314 | 9 | 9 |
|  | *Parus major* |  |  |  |  |  |  | UVS | 314 |  | 25 |
|  | *Pica pica* |  |  |  |  |  |  | VS | 370 |  | 25 |
|  | *Ptilonorhynchus violaceus* | 423 | 514 | 567 | 435 | 534 | 591 | VS | 344 | 15 | 15 |
|  | *Scenopoetes dentirostris* | 424 | 514 | 567 | 438 | 532 | 589 | VS |  | 15 |  |
|  | *Sericulus chrysocephalus* | 418 | 511 | 567 | 431 | 528 | 589 | VS | 349 | 15 | 15 |
|  | *Serinus canaria* | 414 | 506 | 578 | 431 | 531 | 604 | UVS |  | 16 |  |
|  | *Spinus tristis* | 417 | 523 | 579 | 432 | 537 | 596 | VS |  | 17 |  |
|  | *Sturnus vulgaris* | 399 | 515 | 573 | 419 | 536 | 595 | UVS | 337 | 18 | 18 |
|  | *Taeniopygia guttata* | 414 | 510 | 571 | 432 | 537 | 597 | UVS | 321 | 4 | 25 |
|  | *Turdus merula* | 414 | 515 | 570 | 429 | 532 | 593 | UVS | 343 | 13 | 13 |
|  | *Turdus philomelos* |  |  |  |  |  |  | UVS | 335 |  | 25 |
| Podicipediformes | *Podiceps cristatus* |  |  |  |  |  |  | VS | 390 |  | 25 |
| Procellariiformes | *Puffinus pacificus* | 445 | 506 | 562 | 460 | 528 | 586 | VS | 335 | 20 | 20 |
| Psittaciformes | *Melopsittacus undulatus* | 411 | 507 | 566 | 429 | 544 | 592 | UVS | 320 | 4 | 27 |
|  | *Neopsephotus bourkii* |  |  |  |  |  |  | UVS | 334 |  | 25 |
|  | *Platycercus elegans* |  |  |  |  |  |  | UVS | 319 |  | 28 |
| Strigiformes | *Aegolius funereus* |  |  |  |  |  |  | VS | 335 |  | 25 |
|  | *Asio otus* |  |  |  |  |  |  | VS | 356 |  | 25 |
|  | *Athene cunicularia* |  |  |  |  |  |  | VS | 359 |  | 25 |
|  | *Strix aluco* |  |  |  |  |  |  | VS | 353 |  | 25 |
| Struthioniformes | *Rhea americana* | 417 | 506 | 556 | 439 | 524 | 585 | VS |  | 24 |  |
|  | *Struthio camelus* |  |  |  |  |  |  | VS | 369 |  | 24 |

**Table D** Ratio of photoreceptor densities in avian retinas. Photoreceptor with lowest proportion was always given 1.00.

| Order | Species | SWS1 | SWS2 | MWS | LWS | Reference |
| --- | --- | --- | --- | --- | --- | --- |
| Anseriformes | *Anas penelope* | 1.00 | 2.10 | 4.28 | 4.17 | 29 |
|  | *Aythya affinis* | 1.00 | 2.73 | 4.09 | 4.23 | 29 |
|  | *Branta canadensis* | 1.00 | 3.16 | 4.18 | 5.92 | 2 |
| Apodiformes | *Sephanoides sephaniodes* | 1.00 | 2.60 | 4.40 | 3.00 | 3 |
| Charadriiformes | *Anous minutus* | 1.00 | 9.59 | 16.82 | 14.29 | 29 |
|  | *Larus novaehollandiae* | 1.00 | 1.87 | 2.38 | 2.34 | 29 |
| Columbiformes | *Streptopelia chinensis* | 1.00 | 1.25 | 1.61 | 1.43 | 29 |
| Coraciiformes | *Todiramphus sanctus* | 1.00 | 1.32 | 1.55 | 6.36 | 29 |
| Cuculiformes | *Eudynamys scolopacea* | 1.00 | 2.28 | 3.65 | 3.11 | 29 |
| Galliformes | *Gallus gallus* | 1.00 | 1.48 | 2.48 | 2.01 | 30 |
|  | *Pavo cristatus* | 1.00 | 1.88 | 2.20 | 2.11 | 29 |
| Gruiformes | *Gallinula tenebrosa* | 1.00 | 1.69 | 2.10 | 2.19 | 29 |
| Passeriformes | *Ailuroedus crassirostris* | 1.00 | 1.79 | 2.59 | 2.07 | 15 |
|  | *Chlamydera nuchalis* | 1.00 | 1.84 | 2.84 | 2.94 | 15 |
|  | *Entomyzon cyanotis* | 1.00 | 1.96 | 2.70 | 2.61 | 29 |
|  | *Leiothrix lutea* | 1.00 | 2.50 | 2.50 | 5.50 | 12 |
|  | *Manorina melanocephala* | 1.00 | 1.84 | 2.26 | 2.30 | 29 |
|  | *Parus caeruleus* | 1.00 | 1.89 | 2.67 | 2.67 | 29 |
|  | *Parus caeruleus* | 1.00 | 1.92 | 2.68 | 2.70 | 13 |
|  | *Ptilonorhynchus violaceus* | 1.00 | 2.36 | 3.53 | 3.15 | 29 |
|  | *Ptilonorhynchus violaceus* | 1.00 | 1.69 | 2.88 | 3.12 | 15 |
|  | *Scenopoeetes dentirostris* | 1.00 | 2.40 | 3.09 | 2.77 | 15 |
|  | *Sericulus chrysocephalus* | 1.00 | 1.71 | 3.71 | 3.93 | 15 |
|  | *Spinus tristis* | 1.00 | 2.18 | 2.36 | 1.94 | 17 |
|  | *Sturnus vulgaris* | 1.00 | 1.36 | 3.70 | 3.77 | 29 |
|  | *Turdus merula* | 1.00 | 1.71 | 2.14 | 1.89 | 29 |
|  | *Turdus merula* | 1.00 | 1.78 | 2.21 | 1.96 | 13 |
| Pelecaniformes | *Phalacrocorax varius* | 1.00 | 2.45 | 5.83 | 1.43 | 29 |
| Procellariiformes | *Puffinus pacificus* | 1.47 | 1.00 | 1.53 | 2.12 | 29 |
| Psittaciformes | *Cacatua roseicapilla* | 1.00 | 1.24 | 3.96 | 4.18 | 29 |
|  | *Melopsittacus undulatus* | 1.00 | 1.89 | 2.94 | 2.48 | 29 |
|  | *Platycercus eximius* | 1.00 | 1.88 | 3.60 | 3.87 | 29 |
|  | *Trichoglossus chlorolepidotus* | 1.00 | 1.73 | 3.29 | 3.11 | 29 |
|  | *Trichoglossus haematodus* | 1.00 | 1.28 | 2.86 | 2.63 | 29 |

Reference details at end of Supplementary material

1 Jane and Bowmaker 1988 J Comp Physiol A 162:225-235; 2 Moore et al. 2012 J Exp Biol 215:3442-3452; 3 Herrera et al. 2008 J Comp Physiol A 194:785-794; 4 Bowmaker et al 1997 Vis Res 37:2183-2194; 5 Bowmaker et al 1993 Vis Res 33:571-578; 6 Hart et al 1999 Vis Res 39:3321-3328; 7 Hart 2002 J Exp Biol 205:3925-3935; 8 Porter et al. 2014 J Exp Biol 217:3883-3890; 9 Hart et al 2000 J Comp Physiol A 186:681-694; 10 Bowmaker 1977 Vis Res 17:1129-1138; 11 Beason and Loew 2008 Vis Res 48:1-8; 12 Maier and Bowmaker 1993 J Comp Physiol A 172:295-301; 13 Hart et al. 2000 J Comp Physiol A 186:375:387; 14 Hart and Hunt 2007 Am Nat 169:S7-S27; 15 Coyle et al 2012 J Exp Biol 215:1090-1105; 16 Das et al 1999 Vis Res 39:2801-2815; 17 Baumhardt et al 2012 Brain Behav and Evol 83:181-198; 18 Hart et al 1998 J Exp Biol 201:1433-1446; 19 Yokoyama et al 2000 PNAS 97:7366-7371; 20 Hart 2004 J Exp Biol 207:1229-1240; 21 Knott et al 2013 J Exp Biol 216: 4454-4461; 22 Bowmaker and Martin 1985 J Comp Physiol A 156:71-77; 23 Bowmaker and Martin 1978 Vis Res 18:1125-1130; 24 Wright and Bowmaker 2001 Vis Res 41:1-12; 25 Lind et al 2014 J Exp Biol 216:1819-1827; 26 Hastad et al 2009 J Comp Phys A 195:585-590; 27 Lind and Kelber 2009 Vis Res 49:1939-1947; 28 Carvalho et al 2011 Proc Roy Soc 278:107-114; 29 Hart 2001 J Comp Physiol A 187:685-698; 30 Kram et al 2010 PLoS one 5:e8992

**Table E** Summary of photoreceptor sensitivities of the four avian cone classes separated by eye type. Values were derived by compiling all known measurements of these parameters (See Table S2 for complete data and references).

| Cone class | Eye type | Minimum *λ*max (nm) | Maximum *λ*max (nm) | Mean ± SD *λ*max (nm) | N |
| --- | --- | --- | --- | --- | --- |
|  |  |  |  |  |  |
| SWS1 | UVS | 355 | 373 | 368 ± 6 | 14 |
|  | VS | 399 | 424 | 410 ± 8 | 14 |
|  |  |  |  |  |  |
| SWS2 | UVS | 427 | 454 | 444 ± 7 | 13 |
|  | VS | 442 | 463 | 452 ± 6 | 17 |
|  |  |  |  |  |  |
| MWS | UVS | 499 | 509 | 503 ± 3 | 14 |
|  | VS | 497 | 512 | 505 ± 4 | 16 |
|  |  |  |  |  |  |
| LWS | UVS | 557 | 568 | 564 ± 3 | 14 |
|  | VS | 543 | 580 | 566 ± 9 | 17 |
|  |  |  |  |  |  |

**Table F** Summary of oil droplet cut-off parameters of three avian cone classes and T50 of the ocular media, separated by eye type. Values were derived by compiling all known measurements of these parameters (See Table S3 for complete data and references).

| Parameter | Eye type | min λ (nm) | max λ (nm) | mean λ ± SD (nm) | N |
| --- | --- | --- | --- | --- | --- |
|  |  |  |  |  |  |
| OM T50 | UVS | 314 | 344 | 324 ± 11 | 15 |
|  | VS | 335 | 394 | 360 ± 17 | 24 |
|  |  |  |  |  |  |
| λ cut C | UVS | 392 | 423 | 413 ± 9 | 12 |
|  | VS | 417 | 449 | 433 ± 13 | 15 |
|  |  |  |  |  |  |
| Bmid C | UVS | 0.0187* | 0.0273* |  |  |
|  | VS | 0.0380† | 0.0318* |  |  |
|  |  |  |  |  |  |
| λ cut Y | UVS | 502 | 516 | 510 ± 4 | 12 |
|  | VS | 505 | 523 | 512 ± 6 | 16 |
|  |  |  |  |  |  |
| Bmid Y | UVS | 0.0294‡ | 0.0228* |  |  |
|  | VS | 0.0256* | 0.0380† |  |  |
|  |  |  |  |  |  |
| λ cut R | UVS | 561 | 578 | 570 ± 5 | 12 |
|  | VS | 556 | 586 | 567 ± 8 | 16 |
|  |  |  |  |  |  |
| Bmid R | UVS | 0.0294‡ | 0.0187* |  |  |
|  | VS | 0.0170* | 0.0190* |  |  |
|  |  |  |  |  |  |

* Calculated from b

† Obtained from literature

‡ Calculated from λcut and λmid

**Table G** Photoreceptor density ratios values selected for comparisons from all known ratios with the justification for including these in our analyses (See Table S4 for complete data and references).

| Order | Species | SWS1 | SWS2 | MWS | LWS | Reason for inclusion |
| --- | --- | --- | --- | --- | --- | --- |
|  |  |  |  |  |  |  |
| Anseriformes | *Branta canadensis* | 1.00 | 3.16 | 4.18 | 5.92 | Largest SWS2 value  (Excluding *A. minutus*) |
| Charadriiformes | *Anous minutus* | 1.00 | 9.59 | 16.82 | 14.29 | Most skewed overall |
| Columbiformes | *Streptopelia chinensis* | 1.00 | 1.25 | 1.61 | 1.43 | Smallest (Max/Mean) |
| Coraciiformes | *Todiramphus sanctus* | 1.00 | 1.32 | 1.55 | 6.36 | Largest (Max/Mean)  (Excluding *A. minutus*) |
| Galliformes | *Pavo cristatus* | 1.00 | 1.88 | 2.20 | 2.11 | Classic visual system |
| Passeriformes | *Leiothrix lutea* | 1.00 | 2.50 | 2.50 | 5.50 | Most skewed Passerine |
| Passeriformes | *Average* | 1.00 | 1.91 | 2.79 | 2.96 | Average passerine |
| Pelecaniformes | *Phalacrocorax varius* | 1.00 | 2.45 | 5.83 | 1.43 | Most skewed MWS |
| Procellariiformes | *Puffinus pacificus* | 1.00 | 0.68 | 1.04 | 1.44 | SWS2 not SWS1  is smallest value |
|  |  |  |  |  |  |  |

**Table H** Summary of mean dichromatism score changes of 70 species of the Order Galliformes when comparing the influence of changing light environments. Values reported describe the correlation between the scores (Pearson’s r), how many species (out of 70) were assigned the same rank in the comparison (Equal rank), the average rank change (Rank change), the standard deviation of rank change (Rank SD), and the maximum rank change (Max change). The simulations were conducted using both the ‘average UVS’ and ‘average VS’ eye types. See Methods and Results section for more details. Main comparisons are in relation to an ideal illuminant (wavelength independent) and final comparison presents the largest pariwise differences, based on the lowest Pearson’s r value.

| *UVS*  *Eye type* | Pearson’s r | Equal rank | Rank change | Rank  SD | Max change |  | *VS*  *Eye type* | Pearson’s r | Equal rank | Rank change | Rank  SD | Max change |
| --- | --- | --- | --- | --- | --- | --- | --- | --- | --- | --- | --- | --- |
|  |  |  |  |  |  |  |  |  |  |  |  |  |
| Forest Shade | 0.9997 | 41 | 0.74 | 1.18 | 6 |  | Forest Shade | 0.9995 | 33 | 0.83 | 1.23 | 8 |
| Woodland | 0.9997 | 44 | 0.63 | 1.05 | 6 |  | Woodland | 0.9998 | 49 | 0.43 | 0.75 | 3 |
| Blue Sky | 0.9999 | 52 | 0.31 | 0.58 | 2 |  | Blue Sky | 0.9999 | 59 | 0.17 | 0.42 | 2 |
| D65 | 0.9991 | 30 | 1.03 | 1.57 | 10 |  | D65 | 0.9994 | 36 | 0.77 | 1.23 | 8 |
| Gaps | 0.9996 | 36 | 0.80 | 1.12 | 6 |  | Gaps | 0.9996 | 35 | 0.74 | 0.97 | 5 |
| Cloudy | 0.9997 | 42 | 0.69 | 1.10 | 6 |  | Cloudy | 0.9997 | 45 | 0.48 | 0.76 | 3 |
|  |  |  |  |  |  |  |  |  |  |  |  |  |
| Ideal vs D65 | 0.9991 | 30 | 1.03 | 1.57 | 10 |  | Ideal vs D65 | 0.9994 | 36 | 0.77 | 1.23 | 8 |
|  |  |  |  |  |  |  |  |  |  |  |  |  |

**Table I** Summary of mean dichromatism score changes of 70 species of the Order Galliformes when comparing the influence of changing maximum photoreceptor sensitivity values. Values reported describe the correlation between the scores (Pearson’s r), how many species (out of 70) were assigned the same rank in the comparison (Equal rank), the average rank change (Rank change), the standard deviation of rank change (Rank SD), and the maximum rank change (Max change). The simulations were conducted using both the ‘average UVS’ and ‘average VS’ eye types and modifying the photoreceptor sensitivity parameter using the values presented in Table S6. See Methods and Results section for more details. Main comparisons are in relation to the average visual system and final comparison presents the largest pariwise differences, based on the lowest Pearson’s r value.

| *UVS*  *Eye type* | Pearson’s r | Equal score | Rank change | Rank  SD | Max change |  | *VS*  *Eye type* | Pearson’s r | Equal score | Rank change | Rank  SD | Max change |
| --- | --- | --- | --- | --- | --- | --- | --- | --- | --- | --- | --- | --- |
|  |  |  |  |  |  |  |  |  |  |  |  |  |
| SWS1 Max | 0.9999 | 55 | 0.29 | 0.62 | 3 |  | SWS1 Max | 0.9997 | 37 | 0.63 | 0.80 | 3 |
| SWS1 Min | 0.9995 | 48 | 0.46 | 0.83 | 4 |  | SWS1 Min | 0.9997 | 44 | 0.60 | 0.94 | 4 |
| SWS2 Max | 0.9999 | 64 | 0.08 | 0.28 | 1 |  | SWS2 Max | 0.9999 | 53 | 0.34 | 0.70 | 3 |
| SWS2 Min | 0.9996 | 47 | 0.37 | 0.57 | 2 |  | SWS2 Min | 0.9999 | 61 | 0.14 | 0.39 | 2 |
| MWS Max | 0.9999 | 55 | 0.23 | 0.46 | 2 |  | MWS Max | 1.0000 | 65 | 0.09 | 0.33 | 2 |
| MWS Min | 1.0000 | 70 | 0.00 | 0.30 | 0 |  | MWS Min | 1.0000 | 61 | 0.17 | 0.48 | 2 |
| LWS Max | 0.9995 | 48 | 0.46 | 0.81 | 4 |  | LWS Max | 0.9996 | 37 | 0.57 | 0.69 | 3 |
| LWS Min | 0.9994 | 43 | 0.66 | 1.05 | 4 |  | LWS Min | 0.9991 | 38 | 0.63 | 0.82 | 3 |
| All Max | 0.9999 | 50 | 0.34 | 0.61 | 3 |  | All Max | 0.9993 | 33 | 0.77 | 1.04 | 6 |
| All Min | 0.9992 | 35 | 0.74 | 0.96 | 4 |  | All Min | 0.9988 | 29 | 0.94 | 1.14 | 6 |
|  |  |  |  |  |  |  |  |  |  |  |  |  |
| All Max vs All Min | 0.9986 | 27 | 1.03 | 1.20 | 5 |  | All Max vs All Min | 0.9963 | 17 | 1.54 | 1.71 | 9 |
|  |  |  |  |  |  |  |  |  |  |  |  |  |

**Table J** Summary of mean dichromatism score changes of 70 species of the Order Galliformes when comparing the influence of changing oil droplet cut-off values. Values reported describe the correlation between the scores (Pearson’s r), how many species (out of 70) were assigned the same rank in the comparison (Equal rank), the average rank change (Rank change), the standard deviation of rank change (Rank SD), and the maximum rank change (Max change). The simulations were conducted using both the ‘average UVS’ and ‘average VS’ eye types and modifying the oil droplet cut-off parameter using the values presented in Table S7. See Methods and Results section for more details. Main comparisons are in relation to the average visual system and final comparison presents the largest pariwise differences, based on the lowest Pearson’s r value.

| *UVS*  *Eye type* | Pearson’s r | Equal score | Rank change | Rank  SD | Max change |  | *VS*  *Eye type* | Pearson’s r | Equal score | Rank change | Rank  SD | Max change |
| --- | --- | --- | --- | --- | --- | --- | --- | --- | --- | --- | --- | --- |
|  |  |  |  |  |  |  |  |  |  |  |  |  |
| C Max | 0.9999 | 59 | 0.17 | 0.41 | 2 |  | C Max | 1.0000 | 68 | 0.03 | 0.17 | 1 |
| C Min | 0.9999 | 55 | 0.23 | 0.46 | 2 |  | C Min | 0.9988 | 37 | 0.66 | 0.83 | 3 |
| Y Max | 1.0000 | 63 | 0.11 | 0.36 | 2 |  | Y Max | 0.9999 | 64 | 0.09 | 0.28 | 1 |
| Y Min | 0.9999 | 61 | 0.14 | 0.39 | 2 |  | Y Min | 1.0000 | 66 | 0.06 | 0.23 | 1 |
| R Max | 0.9999 | 59 | 0.17 | 0.42 | 2 |  | R Max | 0.9991 | 35 | 0.71 | 0.92 | 5 |
| R Min | 0.9990 | 32 | 0.77 | 0.92 | 4 |  | R Min | 0.9994 | 43 | 0.46 | 0.67 | 3 |
| All Max | 0.9997 | 55 | 0.26 | 0.56 | 3 |  | All Max | 0.9994 | 35 | 0.69 | 0.84 | 4 |
| All Min | 0.9991 | 31 | 0.77 | 0.90 | 4 |  | All Min | 0.9975 | 26 | 0.89 | 0.88 | 4 |
|  |  |  |  |  |  |  |  |  |  |  |  |  |
| R Min vs All Max | 0.9979 | 27 | 1.00 | 1.13 | 6 |  | R Max vs All Min | 0.9947 | 21 | 1.26 | 1.20 | 5 |
|  |  |  |  |  |  |  |  |  |  |  |  |  |

**Table K** Summary of mean dichromatism score changes of 70 species of the Order Galliformes when comparing the influence of changing ocular media absorbance curves. Values reported describe the correlation between the scores (Pearson’s r), how many species (out of 70) were assigned the same rank in the comparison (Equal rank), the average rank change (Rank change), the standard deviation of rank change (Rank SD), and the maximum rank change (Max change). The simulations were conducted using both the ‘average UVS’ and ‘average VS’ eye types and modifying the ocular media absorption parameter by increments spanning the range of values presented in Table S7. See Methods and Results section for more details. Main comparisons are in relation to the average visual system and final comparison presents the largest pariwise differences, based on the lowest Pearson’s r value.

| *UVS*  *Eye type* | Pearson’s r | Equal rank | Rank change | Rank  SD | Max change |  | *VS*  *Eye type* | Pearson’s r | Equal rank | Rank change | Rank  SD | Max change |
| --- | --- | --- | --- | --- | --- | --- | --- | --- | --- | --- | --- | --- |
|  |  |  |  |  |  |  |  |  |  |  |  |  |
| T314 | 1.0000 | 62 | 0.14 | 0.32 | 1 |  | T335 | 0.9999 | 52 | 0.34 | 0.66 | 3 |
| T334 | 1.0000 | 62 | 0.14 | 0.43 | 2 |  | T375 | 0.9998 | 48 | 0.46 | 0.81 | 4 |
| T344 | 0.9999 | 62 | 0.43 | 0.77 | 4 |  | T395 | 0.9995 | 36 | 0.77 | 1.23 | 8 |
|  |  |  |  |  |  |  |  |  |  |  |  |  |
| T314 vs T344 | 0.9998 | 45 | 0.51 | 0.85 | 4 |  | T335 vs T395 | 0.9991 | 28 | 1.06 | 1.46 | 9 |
|  |  |  |  |  |  |  |  |  |  |  |  |  |

**Table L** Summary of mean dichromatism score changes of 70 species of the Order Galliformes when comparing the influence of changing photoreceptor densities. Values reported describe the correlation between the scores (Pearson’s r), how many species (out of 70) were assigned the same rank in the comparison (Equal rank), the average rank change (Rank change), the standard deviation of rank change (Rank SD), and the maximum rank change (Max change). The simulations were conducted using both the ‘average UVS’ and ‘average VS’ eye types and modifying the photoreceptor density parameter using the values presented in Table S8. See Methods and Results section for more details. Main comparisons are in relation to the average visual system and final comparison presents the largest pariwise differences, based on the lowest Pearson’s r value.

| *UVS*  *Eye type* | Pearson’s r | Equal rank | Rank change | Rank  SD | Max change |  | *VS*  *Eye type* | Pearson’s r | Equal score | Rank change | Rank  SD | Max change |
| --- | --- | --- | --- | --- | --- | --- | --- | --- | --- | --- | --- | --- |
|  |  |  |  |  |  |  |  |  |  |  |  |  |
| *Branta canadensis* | 0.9987 | 22 | 1.14 | 1.31 | 8 |  | *Branta canadensis* | 0.9984 | 25 | 1.17 | 1.37 | 8 |
| *Anous minutus* | 0.9926 | 19 | 2.34 | 2.22 | 11 |  | *Anous minutus* | 0.9943 | 14 | 1.89 | 1.79 | 8 |
| *Average Passerine* | 0.998 | 49 | 0.40 | 0.71 | 3 |  | *Average Passerine* | 0.9994 | 35 | 0.69 | 0.81 | 3 |
| *Leiothrix lutea* | 0.9994 | 34 | 0.80 | 1.16 | 7 |  | *Leiothrix lutea* | 0.9990 | 28 | 1.03 | 1.29 | 7 |
| *Pavo cristatus* | 0.9993 | 40 | 0.60 | 0.89 | 4 |  | *Pavo cristatus* | 0.9991 | 36 | 0.69 | 0.88 | 4 |
| *Phalacrocorax varius* | 0.9955 | 21 | 1.20 | 1.10 | 4 |  | *Phalacrocorax varius* | 0.9959 | 24 | 1.09 | 1.19 | 6 |
| *Puffinus pacificus* | 0.9960 | 16 | 1.34 | 1.28 | 5 |  | *Puffinus pacificus* | 0.9967 | 21 | 1.11 | 1.04 | 4 |
| *Streptopelia chinensis* | 0.9976 | 22 | 1.14 | 1.03 | 4 |  | *Streptopelia chinensis* | 0.9978 | 26 | 1.03 | 1.18 | 7 |
| *Todiramphus sanctus* | 0.9996 | 33 | 0.94 | 1.32 | 6 |  | *Todiramphus sanctus* | 0.9994 | 26 | 0.94 | 1.06 | 4 |
|  |  |  |  |  |  |  |  |  |  |  |  |  |
| *A. minutus vs*  *P. pacificus* | 0.9791 | 8 | 3.11 | 2.87 | 12 |  | *A. minutus vs*  *P. pacificus* | 0.9846 | 6 | 2.63 | 2.07 | 10 |
|  |  |  |  |  |  |  |  |  |  |  |  |  |

**Table M** Summary of mean dichromatism score changes of 70 species of the Order Galliformes when comparing the influence of changing the visual system. Values reported describe the correlation between the scores (Pearson’s r), how many species (out of 70) were assigned the same rank in the comparison (Equal rank), the average rank change (Rank change), the standard deviation of rank change (Rank SD), and the maximum rank change (Max change). The simulations were conducted using the parameters of species for which the physical properties of visual systems have been completely characterized, in addition to the ‘average UVS’ and ‘average VS’ systems. See Methods and Results section for more details. Main comparisons are in relation to the average visual system and final comparison presents the comparison between the ‘average UVS’ and ‘average VS’, as well as the largest pariwise difference, based on the lowest Pearson’s r value.

| *UVS*  *Eye type* | Pearson’s R | Equal score | Rank change | Rank  SD | Max change |  | *VS*  *Eye type* | Pearson’s R | Equal score | Rank change | Rank  SD | Max change |
| --- | --- | --- | --- | --- | --- | --- | --- | --- | --- | --- | --- | --- |
|  |  |  |  |  |  |  |  |  |  |  |  |  |
| *M. undulatus* | 0.9990 | 40 | 0.60 | 0.84 | 3 |  | *G. gallus* | 0.9988 | 30 | 0.94 | 1.10 | 6 |
| *C. caerulus* | 0.9997 | 40 | 0.54 | 0.74 | 3 |  | *P. cristatus* | 0.9995 | 39 | 0.66 | 0.90 | 4 |
| *S. vulgaris* | 0.9993 | 42 | 0.69 | 1.04 | 4 |  | *P. pacificus* | 0.9927 | 17 | 1.86 | 1.67 | 7 |
| *T. merula* | 0.9986 | 34 | 0.80 | 0.94 | 3 |  | *P. violaceus* | 0.9977 | 27 | 0.94 | 1.10 | 6 |
|  |  |  |  |  |  |  |  |  |  |  |  |  |
| Average UVS vs  Average VS | 0.9929 | 15 | 2.34 | 2.66 | 16 |  |  |  |  |  |  |  |
| *P. cristatus* vs  *S. vulgaris* | 0.9881 | 18 | 3.00 | 3.06 | 16 |  |  |  |  |  |  |  |
|  |  |  |  |  |  |  |  |  |  |  |  |  |

**Table M** Summary results of mean dichromatism scores (in just-noticeable-differences) for *Tetraogallus tibetanus*, *Arborophila torqueola*, and *Lophophorus impejanus*, generated though an iteration of all possible combinations of the parameters explored in this study (24 948 iterations per eye type). Values associated with the VS eye type were generated using only the range of parameters for the average VS eye type, and the same applies to the UVS eye type. Values associated with the *Pavo cristatus* visual system result from combining the VS and UVS eye type scores.

| Species | Model values |  | Mean | Range | 95 % CI |
| --- | --- | --- | --- | --- | --- |
|  |  |  |  |  |  |
| *Arborophila torqueola* | VS | 4.40 | 4.71 | 3.00 – 6.82 | 3.41 – 6.32 |
|  | UVS | 4.95 | 5.23 | 3.56 – 7.68 | 3.76 – 7.11 |
|  | *Pavo cristatus* | 5.03 | 4.97 | 3.00 – 7.68 | 3.48 – 6.96 |
|  |  |  |  |  |  |
| *Lophophorus impejanus* | VS | 8.49 | 9.25 | 5.26 – 13.75 | 6.19 – 12.58 |
|  | UVS | 9.25 | 9.97 | 6.12 – 14.77 | 6.79 – 13.93 |
|  | *Pavo cristatus* | 9.90 | 9.61 | 5.26 – 14.77 | 6.50 – 13.34 |
|  |  |  |  |  |  |
| *Tetraogallus tibetanus* | VS | 1.09 | 1.12 | 0.41 – 2.17 | 0.59 – 1.83 |
|  | UVS | 1.60 | 1.70 | 0.78 – 2.81 | 0.91 – 2.55 |
|  | *Pavo cristatus* | 1.29 | 1.41 | 0.41 – 2.81 | 0.60 – 2.47 |
|  |  |  |  |  |  |

**References**

Baumhardt, P. E., Moore, B. A., Doppler, M., and E. Fernández-Juricic, E. 2014. Do American goldfinches see their world like passive prey foragers: A study on visual fields, retinal topography, and sensitivity of photoreceptors. Brain, Behavior and Evolution 83: 181-198.

Beason, R. C., and E. R. Loew. 2008. Visual pigment and oil droplet characteristics of the bobolink (*Dolichonyx oryzivorus*), a new world migratory bird. Vision Research 48: 1-8.

Bowmaker, J. K. 1977. The visual pigments, oil droplets and spectral sensitivity of the pigeon. Vision Research 17: 1129-1138.

Bowmaker, J. K., Kovach, J. K., Whitmore, A. V., and E. R. Loew. 1993. Visual pigments and oil droplets in genetically manipulated and carotenoid deprived quail: a microspectrophotometric study. Vision Research 33: 571-578.

Bowmaker, J. K., and G. R. Martin. 1978. Visual pigments and colour vision in a nocturnal bird, *Strix aluco* (tawny owl). Vision Research 18: 1125-1130.

Bowmaker, J. K., and G. R. Martin. 1985. Visual pigments and oil droplets in the penguin, *Spheniscus humboldti*. Journal of Comparative Physiology A 156: 71-77.

Bowmaker, J. K., Heath, L. A., Wilkie, S. E., and D. M. Hunt. 1997. Visual pigments and oil droplets from six classes of photoreceptor in the retinas of birds. Vision Research 37: 2183-2194.

Carvalho, L. S., Knott, B., Berg, M. L., Bennett, A. T., and D. M. Hunt. 2011. Ultraviolet-sensitive vision in long-lived birds. Proceedings of the Royal Society of London B: Biological Sciences 278: 107-114.

Coyle, B. J., Hart, N. S., Carleton, K. L., and G. Borgia. 2012. Limited variation in visual sensitivity among bowerbird species suggests that there is no link between spectral tuning and variation in display colouration. Journal of Experimental Biology 215: 1090-1105.

Das, D., Wilkie, S. E., Hunt, D. M., and J. K. Bowmaker. 1999. Visual pigments and oil droplets in the retina of a passerine bird, the canary *Serinus canaria*: microspectrophotometry and opsin sequences. Vision Research 39: 2801-2815.

Hart, N. S. 2001. Variations in cone photoreceptor abundance and the visual ecology of birds. Journal of Comparative Physiology A 187: 685-697.

Hart, N. S. 2002. Vision in the peafowl (Aves: *Pavo cristatus*). Journal of Experimental Biology 205: 3925-3935.

Hart, N. S. 2004. Microspectrophotometry of visual pigments and oil droplets in a marine bird, the wedge-tailed shearwater *Puffinus pacificus*: topographic variations in photoreceptor spectral characteristics. Journal of Experimental Biology 207: 1229-1240.

Hart, N. S., and D. M. Hunt. 2007. Avian visual pigments: characteristics, spectral tuning, and evolution. American Naturalist 169: S7-S26.

Hart, N. S., Partridge, J. C., and I. C. Cuthill. 1998. Visual pigments, oil droplets and cone photoreceptor distribution in the European starling (*Sturnus vulgaris*). Journal of Experimental Biology 201: 1433-1446.

Hart, N. S., Partridge, J. C., and I. C. Cuthill. 1999. Visual pigments, cone oil droplets, ocular media and predicted spectral sensitivity in the domestic turkey (*Meleagris gallopavo*). Vision Research 39: 3321-3328.

Hart, N. S., Partridge, J. C., Bennett, A. T. D., and I. C. Cuthill. 2000a. Visual pigments, cone oil droplets and ocular media in four species of estrildid finch. Journal of Comparative Physiology A 186: 681-694.

Hart, N. S., Partridge, J. C., Cuthill, I. C., and A. T. Bennett. 2000b. Visual pigments, oil droplets, ocular media and cone photoreceptor distribution in two species of passerine bird: the blue tit (*Parus caeruleus* L.) and the blackbird (*Turdus merula* L.). Journal of Comparative Physiology A 186: 375-387.

Håstad, O., Partridge, J. C., and A. Ödeen. 2009. Ultraviolet photopigment sensitivity and ocular media transmittance in gulls, with an evolutionary perspective. Journal of Comparative Physiology A 195: 585-590.

Herrera, G., Zagal, J. C., Diaz, M., Fernández, M. J., Vielma, A., Cure, M., Martinez, J., Bozinovic, F.,and A. G. Palacios. 2008. Spectral sensitivities of photoreceptors and their role in colour discrimination in the green-backed firecrown hummingbird (*Sephanoides sephaniodes*). Journal of Comparative Physiology A 194: 785-794.

Jane, S. D., & Bowmaker, J. K. (1988). Tetrachromatic colour vision in the duck (Anas platyrhynchos L.): microspectrophotometry of visual pigments and oil droplets. *Journal of Comparative Physiology A*, *162*(2), 225-235.

Knott, B., Davies, W. I., Carvalho, L. S., Berg, M. L., Buchanan, K. L., Bowmaker, J. K., Bennett, A. T. D. and D. M. Hunt. 2013. How parrots see their colours: novelty in the visual pigments of *Platycercus elegans*. Journal of Experimental Biology 216: 4454-4461.

Kram, Y. A., Mantey, S., and J. C. Corbo. 2010. Avian cone photoreceptors tile the retina as five independent, self-organizing mosaics. PLoS One 5: e8992.

Lind, O., and A. Kelber. 2009. Avian colour vision: Effects of variation in receptor sensitivity and noise data on model predictions as compared to behavioural results. Vision Research 49: 1939-1947.

Lind, O., Mitkus, M., Olsson, P., and A. Kelber. 2013. Ultraviolet sensitivity and colour vision in raptor foraging. Journal of Experimental Biology 216: 1819-1826.

Maier, E. J., and J. K. Bowmaker. 1993. Colour vision in the passeriform bird, *Leiothrix lutea*: correlation of visual pigment absorbance and oil droplet transmission with spectral sensitivity. Journal of Comparative Physiology A 172: 295-301.

Moore, B. A., Baumhardt, P., Doppler, M., Randolet, J., Blackwell, B. F., DeVault, T. L., Loew, E. R., and E. Fernández-Juricic. 2012. Oblique color vision in an open-habitat bird: spectral sensitivity, photoreceptor distribution and behavioral implications. Journal of Experimental Biology 215: 3442-3452.

Porter, M. L., Kingston, A. C., McCready, R., Cameron, E. G., Hofmann, C. M., Suarez, L., Olsen, J. H., Cronin, T. W., and P. R. Robinson. 2014. Visual pigments, oil droplets, lens, and cornea characterization in the whooping crane (*Grus americana*). Journal of Experimental Biology jeb-108456.

Yokoyama, S., Radlwimmer, F. B., and N. S. Blow. 2000. Ultraviolet pigments in birds evolved from violet pigments by a single amino acid change. Proceedings of the National Academy of Sciences 97: 7366-7371.

Wright, M. W., and J. K. Bowmaker. 2001. Retinal photoreceptors of paleognathous birds: the ostrich (*Struthio camelus*) and rhea (*Rhea americana*). Vision Research 41: 1-12.
